# Exercise and tissue fibrosis: recent advances in therapeutic potential and molecular mechanisms

**DOI:** 10.3389/fendo.2025.1557797

**Published:** 2025-03-20

**Authors:** Zheng Zhao, Yongjia Zhu, Dongfeng Wan

**Affiliations:** ^1^ School of Physical Education, Anyang Normal University, Anyang, Henan, China; ^2^ School of Clinical Medicine, Shandong Second Medical University, Weifang, Shandong, China; ^3^ School of Health, Shanghai Normal University Tianhua College, Shanghai, China

**Keywords:** exercise, fibrosis, molecular mechanisms, therapeutics, physicotherapeutics

## Abstract

Tissue fibrosis represents an aberrant repair process, occurring because of prolonged injury, sustained inflammatory response, or metabolic disorders. It is characterized by an excessive accumulation of extracellular matrix (ECM), resulting in tissue hardening, structural remodeling, and loss of function. This pathological phenomenon is a common feature in the end stage of numerous chronic diseases. Despite the advent of novel therapeutic modalities, including antifibrotic agents, these have only modest efficacy in reversing established fibrosis and are associated with adverse effects. In recent years, a growing body of research has demonstrated that exercise has significant benefits and potential in the treatment of tissue fibrosis. The anti-fibrotic effects of exercise are mediated by multiple mechanisms, including direct inhibition of fibroblast activation, reduction in the expression of pro-fibrotic factors such as transforming growth factor-β (TGF-β) and slowing of collagen deposition. Furthermore, exercise has been demonstrated to assist in maintaining the dynamic equilibrium of tissue repair, thereby indirectly reducing tissue damage and fibrosis. It can also help maintain the dynamic balance of tissue repair by improving metabolic disorders, exerting anti-inflammatory and antioxidant effects, regulating cellular autophagy, restoring mitochondrial function, activating stem cell activity, and reducing cell apoptosis, thereby indirectly alleviating tissue. This paper presents a review of the therapeutic potential of exercise and its underlying mechanisms for the treatment of a range of tissue fibrosis, including cardiac, pulmonary, renal, hepatic, and skeletal muscle. It offers a valuable reference point for non-pharmacological intervention strategies for the comprehensive treatment of fibrotic diseases.

## Graphical Abstract

Exercise alleviates tissue fibrosis through multiple-target mechanisms, including inhibiting fibroblast activation, downregulating pro-fibrotic factors such as TGF-β, regulating ECM remodeling, reducing inflammation, enhancing antioxidant capacity, improving mitochondrial function, balancing autophagy and apoptosis, and improving lipid metabolism.

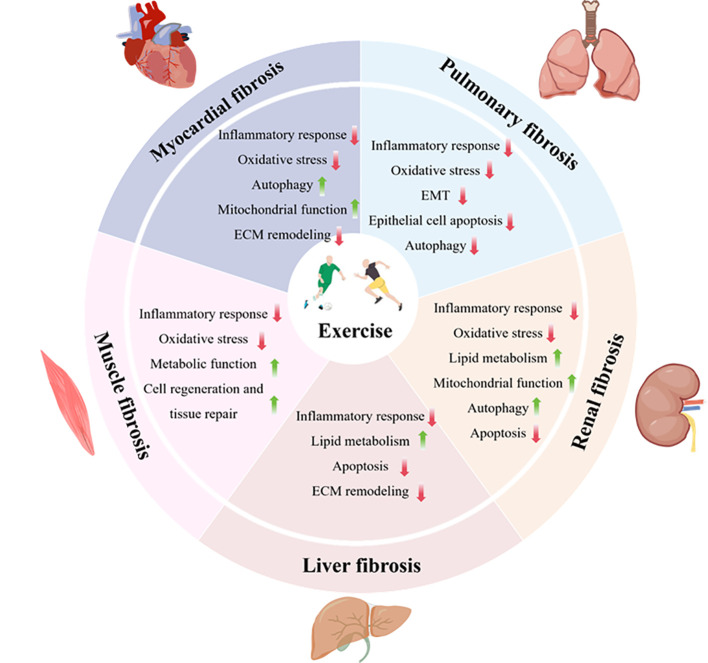

## 1 Introduction

Tissue fibrosis represents an aberrant repair process, precipitated by chronic injury, sustained inflammatory response, or metabolic abnormalities. This manifests as an excessive accumulation of extracellular matrix (ECM), leading to tissue sclerosis, structural remodeling, and loss of function (1). Although tissue fibrosis is essentially a self-repairing response after tissue injury, the proliferating mesenchymal cells and fibrous connective tissues lack the function of the original parenchymal cells. As a result, the function of the organ is not restored. However, if the repair response is too strong, it can lead to dysfunction of tissues and organs or even cause serious consequences, including organ failure and death. Fibrosis is a common phenomenon observed in various organs, including the liver, lungs, heart, kidneys, and skeletal muscle. The pathological mechanisms typically encompass cellular transformation, aberrant extracellular matrix deposition, and cell-cell and cell-matrix interactions (2). The activation of fibroblasts and myofibroblasts, along with the up-regulation of pro-fibrotic factors, such as transforming growth factor-β (TGF-β), represent the key mechanisms involved in the progression of fibrosis, which is a common feature of fibrotic progression in various tissues (3, 4). The etiology of fibrosis is multifaceted, with a plethora of factors implicated in its development. These include viral infections, metabolic disorders, and toxic exposures (such as renal injury, chronic inflammation, mechanical injuries, and autoimmune diseases) (5, 6), which collectively contribute to the gradual loss of organ function through a cycle of continuous damage and repair responses. Epidemiological data indicate that the global prevalence of idiopathic pulmonary fibrosis (IPF) is 3-45 cases per 100,000 individuals, with the highest incidence rates reported in South Korea, Canada, and the United States. Additionally, the mortality rate associated with IPF is on the rise (7). A cross-sectional study comprising over 5.7 million individuals revealed that the prevalence of advanced fibrosis and cirrhosis in Chinese adults was 2.85% and 0.87%, respectively (8). Renal fibrosis, a representative pathological feature of diabetic nephropathy, is particularly prevalent in diabetic patients and a significant cause of mortality in this population (9). Despite recent advances in the treatment of fibrosis (e.g., antifibrotic drugs such as pirfenidone and nintedanib), the currently available therapies remain ineffective in reversing established fibrosis and are often associated with adverse effects. Consequently, the search for more effective and safer treatments is a priority area of antifibrotic research.

It is widely acknowledged that physical activity represents a safe and viable non-pharmacological intervention, offering an effective strategy for the prevention and treatment of a multitude of chronic diseases (10, 11). Exercise interventions are broadly categorized into aerobic exercises (e.g., running, swimming), resistance training (e.g., weightlifting), high-intensity interval training (HIIT) and flexibility and balance exercises (10). In recent years, an increasing number of studies have demonstrated that exercise has significant advantages and potential for combating fibrosis in several tissues. From a physiological perspective, exercise can directly regulate the activation state of fibroblasts, down-regulate the production of pro-fibrotic factors such as TGF-β, inhibit collagen deposition, and slow down or reverse the process of tissue fibrosis (12–14). On the other hand, aerobic exercise, represented by running, can improve metabolic disorders and reduce lipid deposition, particularly in the liver, alleviating tissue fibrosis caused by metabolic disturbances (15, 16). At the same time, both aerobic exercise and HIIT possess strong anti-inflammatory and antioxidant effects. These exercises can lower the levels of inflammatory factors and reactive oxygen species (ROS) in the body, reducing sustained tissue damage and thus inhibiting the activation of pro-fibrotic signaling pathways (16–18). Furthermore, exercise can assist in maintaining the normal dynamic equilibrium of tissue repair by regulating cellular autophagy and restoring mitochondrial function, activating the function of stem cells, and so forth. This promotes normal tissue repair and regeneration while reducing fibrosis-related apoptosis and pathological remodeling (19–22). For example, resistance training promotes muscle hypertrophy and strength via mechanical loading, modulating stem cell activity to attenuate skeletal muscle fibrosis (23). Additionally, different intensities and types of exercise may play distinct roles in fibrosis control. The combined application of multiple exercise modalities has also demonstrated more favorable outcomes in the intervention of multi-organ fibrosis (24). Therefore, tailoring exercise type and intensity to disease context is critical for optimizing antifibrotic outcomes.

Nevertheless, there is currently no comprehensive review of the therapeutic potential of exercise for all types of tissue fibrosis. This paper provides a review of the molecular mechanisms by which exercise ameliorates a range of tissue fibrosis diseases. It is anticipated that the insights and new ways of thinking provided by this review will contribute to a deeper understanding of the pathogenesis of tissue fibrosis and of exercise as a comprehensive treatment for fibrotic diseases.

## 2 The occurrence of tissue fibrosis

Following tissue injury, damaged sites release danger signals that promote the release of pro-inflammatory cytokines by inflammatory cells. Along with cytokine secretion, these cells also produce large amounts of ROS. Elevated ROS levels and sustained inflammation lead to mitochondrial dysfunction and metabolic disorders, further inducing autophagic dysregulation and cell apoptosis. The interaction between inflammation, oxidative stress, mitochondrial dysfunction, autophagic abnormalities, and apoptosis activates fibroblasts, leading to the excessive accumulation of ECM components such as collagen and fibronectin, thereby triggering fibrosis in organs such as the heart, lungs, kidneys, liver, and skeletal muscles (25–27). Tissue fibrosis is regulated by various factors, with TGF-β playing a particularly significant role in the process (28–30). TGF-β, as a multifunctional polypeptide cytokine, can bind to TGF-β receptors, activating downstream Small mother against decapentaplegic proteins (Smad)-dependent signaling pathways. This results in the phosphorylation of Smad2/3, which, after binding with Smad4, enters the cell nucleus and induces tissue fibrosis (31, 32). Additionally, TGF-β can also mediate tissue fibrosis through non-Smad pathways, such as mitogen-activated protein kinase (MAPK) (33). Studies indicate that the activation of the TGF-β signaling pathway plays an essential role in the progression of fibrosis in tissues and organs such as the heart (34), lung (35), kidneys (36), liver (37) and skeletal muscles (38).

Fibrosis occurs in nearly all tissues and organs in the body. Myocardial fibrosis is a common pathological manifestation in cardiovascular diseases such as myocardial infarction (MI) (39), coronary heart disease (40), and diabetic cardiomyopathy (DCM) (41), leading to scar formation, reduced compliance, and ultimately affecting heart function. Pulmonary fibrosis is a chronic lung disease characterized by thickening of the alveolar wall, primarily involving repeated damage to alveolar epithelial cells and the accumulation and activation of fibroblasts (42). IPF, a common form of pulmonary fibrosis, can result in progressive pulmonary failure and poor prognosis (43). Renal fibrosis is the most common pathological change in chronic kidney disease (CKD), characterized by excessive ECM deposition, leading to gradual loss of kidney function, and ultimately progressing to end-stage renal disease (44). Liver fibrosis refers to the diffuse excessive deposition and abnormal distribution of the extracellular matrix in the liver, commonly seen in nonalcoholic fatty liver disease (NAFLD and nonalcoholic steatohepatitis (NASH) (45, 46). Traditionally, liver fibrosis was considered reversible at the histological level, but without intervention, it can progress to cirrhosis, making reversal extremely difficult at that stage (47). Similar to other organs, skeletal muscle fibrosis is also a pathological process characterized by abnormal ECM deposition, resulting in muscle structural changes, stiffness, and decreased muscle strength, ultimately leading to functional impairments in movement (48, 49). It can be caused by various factors, including idiopathic inflammatory myopathies, muscular dystrophies, ischemia-reperfusion, radiation injury, and chronic diseases such as diabetes (50–54). Additionally, aging and prolonged immobilization can lead to a decline in muscle mass, resulting in skeletal muscle atrophy and fibrosis (50–54).

Fibrosis is a necessary process for tissue repair following injury, but persistent or severe fibrosis can lead to the destruction of tissue structure, ultimately resulting in organ failure (26). Therefore, it is essential to further elucidate the mechanisms underlying tissue fibrosis and develop effective treatment strategies.

## 3 Regulation of myocardial fibrosis by exercise

### 3.1 Exercise and myocardial fibrosis

A substantial body of evidence from numerous studies has demonstrated the therapeutic benefits of exercise in the treatment of myocardial fibrosis of multiple etiologies. The results of a study has demonstrated that patients with endomyocardial fibrosis (EMF) who engage in exercise three times a week, with each session consisting of 40 minutes of aerobic exercise and 20 minutes of strength training, for a total duration of 4 months of combined aerobic and resistance training, can improve their maximal oxygen uptake, myocardial function, and quality of life (55). A previous study demonstrated that 12 weeks of moderate-intensity aerobic exercise, comprising 30 minutes of exercise three times per week, led to an improvement in peak oxygen uptake in patients with heart failure (HF) who exhibited low galectin-3 expression in their blood. However, this improvement was less pronounced in patients with elevated galectin-3 levels. This indicates that the beneficial effects of exercise may be mediated by fibrosis (56). Additionally, other study has identified fibrotic deposition of extracellular matrix as a potential contributor to myocardial stiffness in patients with HF with preserved ejection fraction (HFpEF) (57). Hieda et al. (58) observed that sustained exercise over one year improved myocardial stiffness in stage B HFpEF patients with myocardial stiffness, suggesting that exercise may improve myocardial fibrosis (**Table 1**).

**Table 1:** Clinical study of exercise intervention in tissue fibrosis.

Organ	Population	Exercise protocol	Outcome	Reference
Heart	EMF patients	40 min of aerobic exercise combine 20 min of strength training, 3 days/week, 4 months	functional capacity↑ cardiac volumes↑ quality of life↑	(55)
	HF patients	aerobic exercise , 35 min/day, 3 days/week, 12 weeks	peak oxygen uptak↑	(56)
	stage B HFpEF patients	Individualized training methods included high-intensity aerobic intervals, lower intensity endurance training, and strength training, 1 year	LV myocardial stiffness↓	(58)
Lung	ILDs patients	30 min of aerobic exercise, cycling and walking, plus upper and lower limb resistance training, 2 days/week, 8 weeks	6 MWD↑ health-related quality of life↑ dyspnoea↓	(92)
	IPF patients	60-minute supervised group exercise training, 2 days/week, 12weeks	cardiovascular function↑ functional capacity ↑ quality of life↑ Dyspnea ↓	(93)
Kidney	stage III diabetic nephropathy patients	more than 300 min of physical activity per week	BMI↓ HbA1c ↓ Urine ACR↓ Serum/Uurine 8-OHdG↓ plasma TGF-β1↓ serum HDL-C ↑ serum SOD ↑ serum MDA↓	(112)
Liver	NASH patients	moderate-intensity aerobic exercise sessions (45–55% VO2peak), each lasting 30 min, 5 days/week, 12weeks	serum FGF21↓	(147)
	MetS patients	150 minutes of moderate-intense physical activity per week, 6 months	liver transaminases ↓ alkaline liver phosphatase ↓ liver γ-glutamyl transferase ↓ aspartate aminotransferase-to-platelet ratio index score ↓	(148)
	NAFLD patients	moderate to vigorous aerobic exercise, 30 to 60 minutes each time, 3 to 4 times/week, 2 years	FLI ↓ NAFLD-FS ↓	(149)
	NASH patients	aerobic (cycling) and resistance training, 3 days/week, 12 weeks	HTGC↓ visceral fat↓ plasma triglycerides↓ plasma γ-glutamyltransferase↓	(150)
Skeletal muscle	myositis patients	resistance exercise, with an intensity of 10 voluntary repetition maximum, 3 days/week, 7 weeks	muscle strength↑ VO2max↑ serum C1q ↓ proinflammatory and profibrotic gene expression↓ antiinflammatory andantifibrotic gene expression↑	(176)
	olderly healthy male	One-time acute resistance training: concentric-eccentric knee extension exercise, concentric-eccentric knee extension exercise followed by foam rolling, eccentric knee extension exercise, plyometric exercise.	activity of genes associated with ECM remodeling(MMP3,MMP9,TIMP1)↑	(178)

↓, means DOWN; ↑, means UP.

### 3.2 Mechanisms of exercise modulation of myocardial fibrosis

#### 3.2.1 Inflammatory response

MI represents one of the most prevalent primary diseases in which remodeling of the heart and myocardial fibrosis occur. In the aftermath of severe MI, patients frequently endure irreversible myocardial damage and fibrosis. Furthermore, chronic inflammation represents a significant contributing factor in the development of myocardial fibrosis (59). It has been demonstrated that the intraperitoneal administration of 10 mg/kg MCC950, an NLR family pyridine structure domain 3 (NLRP3) inflammatory vesicle inhibitor, resulted in the inhibition of collagen type I alpha 1 chain (COL1A1), collagen type III alpha 1 chain (COL3A1) and α-Smooth muscle actin (α-SMA) expression in the myocardium of mice with MI. Additionally, *in vitro* experiments demonstrated that MCC950 could suppress the expression of COL1A1 and α-SMA in cardiac fibroblasts (CFs) (60). This indicates that the inhibition of inflammation may prove an effective method of improving myocardial fibrosis. DCM is a distinctive pathological condition that affects individuals with diabetes. Myocardial fibrosis represents the most prominent histopathological alteration in DCM (61). It has been demonstrated that aerobic exercise can act as an anti-inflammatory agent to some extent and play an active role in combating myocardial fibrosis due to an inflammatory response. A recent study observed that mice subjected to treadmill exercise at a speed of 7-12 m/min for 30-40 minutes, five days a week for eight weeks, exhibited a downregulation of the expression levels of the fibrosis markers COL1A1, COL3A1, and α-SMA in the myocardium of DCM mice, accompanied by an inhibition of the activation of the TGF-β1/Smad pathway. Furthermore, the expression of inflammatory proteins, including Tumor necrosis factor-α (TNF-α), Interleukin-6 (IL-6),Phosphorylation-Inhibitor of Nuclear factor kappa-κB (p-IκB)α/-IκBα, and Phosphorylation-Nuclear factor kappa-κB p65 (p-NF-κB p65)/NF-κB p65, was reduced (62). Chen et al. (63) also demonstrated that aerobic exercise could alleviate myocardial inflammation and fibrosis in rats on high-fat diets by inhibiting the P2X7 purinergic receptor. Furthermore, in women, postmenopausal estrogen deficiency and hypertension may act synergistically to induce myocardial inflammation and promote myocardial fibrosis. However, the precise mechanisms involved remain unclear. The animal experiments conducted by Lin et al. (64) substantiated that running table exercise exerts a therapeutic improvement effect on cardiac inflammation and myocardial fibrosis due to this cause.

#### 3.2.2 Oxidative stress

The current evidence base indicates that oxidative stress plays a significant role in the pathogenesis of myocardial fibrosis (65, 66). It was observed that the overexpression of manganese superoxide dismutase (Mn isoform of superoxide dismutase, MnSOD) resulted in the suppression of COL1A1 and TGF-β expression, which are markers of fibrosis, in the myocardium of aged mice (67). A recent study demonstrated that swimming exercise preconditioning twice a day for 90 minutes over three weeks resulted in the suppression of COL1A1, COL3A1 and TGF-β in the myocardium of mice with HF induced by transverse aortic constriction (TAC) (68). The *in vitro* results demonstrated that a 1μmol/L norepinephrine (NE) intervention enhanced the expression of fibrosis-associated mRNA and protein levels in CFs. Conversely, pretreatment of cardiac hypertrophy induced by swimming exercise promoted senescence, increased apoptosis, and inhibited the proliferation of CFs *in vitro (* 68). On this basis, the knockdown of NF-E2-related factor-2 (Nrf2) was observed to prevent the inhibitory effect of exercise preconditioning on fibrosis. This suggests that exercise inhibits myocardial fibrosis in HF mice by modulating Nrf2 (68). Jia et al. (69) observed that four weeks of exercise training resulted in a down-regulation of ROS production and an up-regulation of superoxide dismutase 1(SOD1) and SOD2 expression in the myocardium of MI rats, thereby inhibiting myocardial fibrosis. Another study demonstrated that swimming exercise for 15 minutes per day, 5 days per week for 8 weeks inhibited myocardial fibrosis, apoptosis, and ROS production, and upregulated Sirtuins3 (SIRT3) expression and antioxidant enzyme SOD2 activity in aged MI mice (70). Furthermore, the knockdown of SIRT3 in aged cardiomyocytes exacerbated ischemia-induced apoptosis and ROS production. This suggests that exercise inhibits cardiomyocyte apoptosis and ROS production by promoting SIRT3 expression, thereby attenuating myocardial fibrosis (70).

#### 3.2.3 Autophagy

Autophagy is a process by which cells degrade and recycle intracellular components to maintain intracellular homeostasis (71). It is widely acknowledged that exercise plays an important role in regulating myocardial autophagy (72, 73). For instance, Guo et al. (19) demonstrated that resistance exercise at 65%-70% of peak oxygen uptake for 60 minutes per day A regimen of five days per week, for eight weeks, inhibited myocardial fibrosis and promoted light chain 3-I (LC3I), LC3II and mitochondrial autophagy PTEN-induced putative kinase1 (PINK1) in HF mice, as well as Parkin protein expression and downregulating P62 protein expression (19). It was demonstrated that Doxorubicin (DOX) induced cardiotoxicity in mice exhibited a reduction in myocardial fibrosis, Uncoupling Protein 2 (UCP2), endothelial markers (CD31 and E-cadherin), and autophagy proteins (Beclin-1 and LC3II/LC3I) expression, while mesenchymal markers (α-SMA and Vimentin) and p62 expression were increased (74). The administration of running table exercise at a velocity of 13 m/min for 60 minutes per day, 5 days per week, for a total of 6 weeks resulted in the reversal of these phenomena. Irisin, a cleavage product of its precursor fibronectin type III domain-containing 5 (FNDC5), is an exercise-induced muscle factor (75). It is regarded as a crucial mediator of the exercise-induced effects. *In vitro* experimental results indicate that treatment with 20 nM irisin can ameliorate DOX-induced dysfunction of cardiac microvascular endothelial cells (CMECs) by inhibiting endothelial-to-mesenchymal transition (EndMT) (74). Furthermore, the pretreatment of CMECs with irisin was observed to inhibit ROS production, NF-κB pathway activation, and EndMT. Subsequent a study demonstrated that autophagy-mediated the inhibition of ROS by irisin, thereby ameliorating DOX-induced EndMT. Conversely, the knockdown of UCP2 in CMEC cells was found to inhibit the effects of irisin. This indicates that exercise increases UCP2 expression by stimulating irisin production, which in turn enhances autophagy within cells and consequently reduces ROS production. Furthermore, it downregulates the NF-κB signaling pathway, thereby inhibiting EndMT and consequently ameliorating myocardial fibrosis in mice (74).

#### 3.2.4 Mitochondrial function

Up to now, numerous studies have demonstrated that mitochondrial dysfunction is a key factor in the development of myocardial fibrosis (76, 77). The SIRT family has been identified as a crucial regulator of mitochondrial homeostasis, with a significant impact on the progression of myocardial fibrosis (78). Several studies have demonstrated that aerobic exercise can significantly attenuate MI-induced mitochondrial damage and improve mitochondrial function. Furthermore, a study by Zhao et al. (70) found that short-duration (15 min) swimming exercise significantly improved left ventricular function and survival, and suppressed myocardial fibrosis and apoptosis in older mice in the stable phase after MI, as compared with long-duration exercise (60 min). From a mechanistic perspective, short-duration swimming exercise was observed to reduce the disturbance of mitochondrial dynamics and promote mitochondrial autophagy. This was achieved by increasing the level of SIRT3 protein and improving mitochondrial quality control, which in turn led to an improvement in mitochondrial function in cardiomyocytes and a subsequent inhibition of myocardial fibrosis progression (70). Furthermore, evidence indicates that resistance exercise can enhance myocardial mitochondrial autophagic activity through the activation of the HIF1α-Parkin pathway, which has been demonstrated to have a protective effect on myocardial morphology and myocardial fibrosis in mice with chronic HF (19). It is noteworthy that the combination of moderate-intensity continuous training with resistance training exhibited superior protection compared to the resistance training model (19). Jia et al. (69) reported that four weeks of platform training was sufficient to significantly improve left ventricular function and reduce the markers of cardiac fibrosis. Subsequent a study has identified the adaptive activation of the SIRT1/Peroxisome proliferator-activated receptor gamma coactivator 1-α (PGC-1α)/Phosphoinositide 3-kinase (PI3K)/Protein kinase B (Akt) signaling pathway in myocardial tissues following MI (69). Furthermore, exercise training has been demonstrated to enhance the activation of the SIRT1/PGC-1α/PI3K/Akt signaling pathway, thereby improving mitochondrial integrity, and promoting biosynthesis, consequently, mediating exercise-induced cardioprotection following MI (69). A recent study demonstrated that resistance exercise also promotes FNDC5 expression, activates the AMPK-SIRT1 pathway, and inhibits myocardial fibrosis in mice with MI (17). *In vitro*, findings demonstrated that irisin treatment inhibited the H2O2-induced activation of the TGF-β1/Smad2/3 signaling pathway and the expression of fibrosis-related markers, including connective tissue growth factor (CTGF), α-SMA, COL1A1, and COL3A1, in CFs (17). Furthermore, the knockdown of SIRT1 was observed to exacerbate the effect of H2O2. This suggests that resistance exercise inhibits MI myocardial fibrosis by promoting irisin expression through the activation of the AMP-activated protein kinase (AMPK)-SIRT1 pathway (17).

#### 3.2.5 ECM remodeling

ECM is a complex three-dimensional network structure composed of various macromolecules, primarily including collagen, elastin, glycoproteins (such as fibronectin and laminin), proteoglycans, and glycosaminoglycans (79, 80). ECM not only provides structural support and mechanical stability to cells but also participates in processes such as cell adhesion, migration, proliferation, and differentiation. Through interactions with cell surface receptors, ECM activates multiple signaling pathways, thereby regulating cell fate and function (79, 80). As a highly dynamic structure, ECM remodeling plays a critical role in tissue development, repair, regeneration, and the maintenance of tissue and organ homeostasis (81). In pathological conditions, however, an imbalance in ECM remodeling is often the ultimate pathological manifestation of tissue fibrosis and is the most direct mechanism driving its occurrence (82). The dynamic balance and remodeling of ECM rely on the precise regulation of various proteases, including matrix metalloproteinases (MMPs), Adamalysins (including ADAMs and ADAMTS), and tissue inhibitors of metalloproteinases (TIMPs) (83). Disruption in their balance is a key trigger for ECM deposition. Exercise has been shown to regulate the function of MMPs, TIMPs, and other factors, inhibiting the excessive synthesis of ECM components while promoting ECM degradation, thereby restoring or maintaining the dynamic balance of ECM and effectively improving fibrosis. Among the MMPs family, MMP2 and MMP9 are two important members associated with myocardial fibrosis regulation (84). Abnormal activation of these enzymes often leads to increased collagen levels and exacerbated fibrosis (84). A study has reported that moderate aerobic exercise significantly downregulated the expression of MMP2 and MMP9 proteins in myocardial tissue after acute myocardial infarction in rats, while promoting the expression of their inhibitor TIMP2, effectively improving the degree of myocardial fibrosis and alleviating ventricular remodeling after myocardial infarction (85). At the molecular level, Ma et al. (86) confirmed that exercise training increases the expression of fibroblast growth factor 21 (FGF21) protein, inhibits the activation of the TGF-β1-Smad2/3-MMP2/9 signaling pathway, and reduces collagen synthesis, ultimately alleviating cardiac fibrosis and improving cardiac dysfunction in mice after myocardial infarction. MicroRNA (miRNA) is a conserved non-coding RNA that plays a broad role in regulating gene expression in eukaryotes by interfering with transcription, translation, or epigenetic processes (87). MiRNAs can act as active molecules in exosomes released by cardiomyocytes, facilitating information transfer. For example, exercise can promote the release of exosomes containing high levels of miR-455, miR-29b, miR-323-5p, and miR-466 by cardiomyocytes, and the miRNAs in these exosomes can inhibit MMP-9 activity and block ECM remodeling, thereby reducing myocardial fibrosis in db/db mice (88). Myocardial fibrosis and ECM deposition are also key features of cardiac dysfunction in aging. A Study has shown that exercise can improve the MMP and TIMP imbalance associated with aging, thereby improving age-related myocardial fibrosis (89). Interestingly, in Fischer 344 × Brown Norway F1 (FBNF1) rats, the levels of active MMP-1, active MMP-2, and MMP-14 in the left ventricular ECM decrease with age, while TIMP-1 levels increase (89). Twelve weeks of endurance training reversed these changes, alleviated collagen accumulation and ECM remodeling in the aging heart, and attenuated age-related myocardial fibrosis (89).

Despite the plethora of evidence attesting to the substantial beneficial impact of exercise on myocardial fibrosis, However, recent studies have also indicated that inappropriate exercise may potentially exacerbate myocardial fibrosis. A study conducted on animals revealed that following 16 weeks of high-intensity strenuous exercise, the rats in the experimental group exhibited myocardial hypertrophy, atrial dilatation, and cardiac fibrosis, in addition to a decline in ventricular function and an increase in the incidence of arrhythmias (90). Furthermore, a study by Maria et al. also identified the presence of myocardial fibrosis and impaired systolic and diastolic function in the right ventricle following prolonged high-intensity exercise. However, the function of the left ventricle was not significantly affected, and moderate-intensity exercise did not result in the formation of myocardial fibrosis in the right ventricle (91).

**Figure 1:**
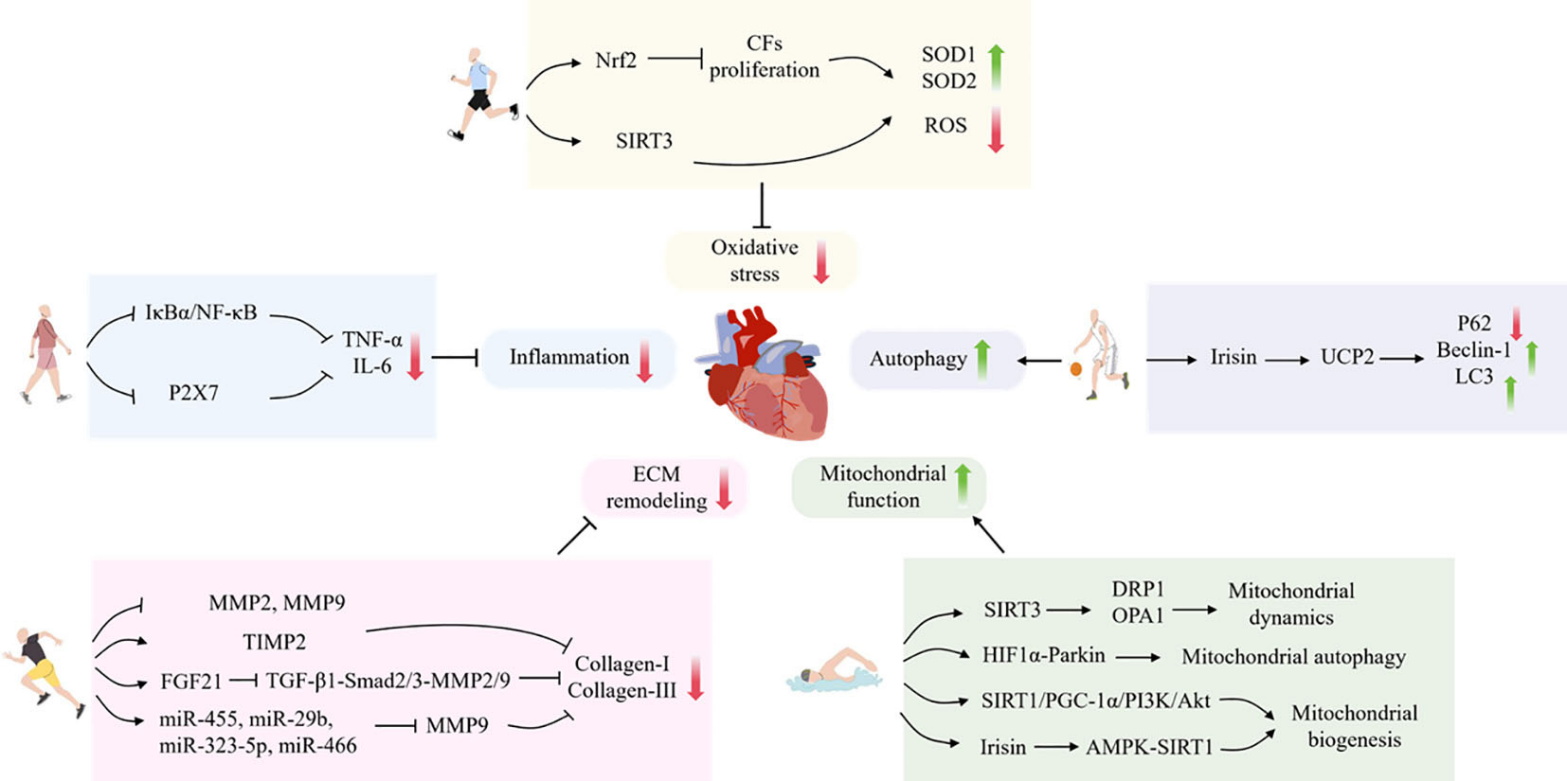
The mechanism by which exercise protects against myocardial fibrosis.

In conclusion, the processes of inflammation, oxidative stress, autophagy, mitochondrial function and ECM remodeling are closely related to the process of myocardial fibrosis. It has been demonstrated that exercise training, as a non-pharmacological treatment, can improve myocardial fibrosis by regulating the aforementioned processes. However, some studies have indicated that long-term high-intensity exercise training may, conversely, result in damage to cardiomyocytes, induce or accelerate myocardial fibrosis, and, in severe cases, may even precipitate consequences such as sudden cardiac death. Therefore, it is of paramount importance to select the appropriate type of exercise (**Figure 1**) (**Table 2**).

**Table 2:** Preclinical study of exercise intervention in tissue fibrosis.

Organ	Model	Exercise protocol	Effects and related mechanisms	Reference
Heart	db/db mice	Treadmill exercise: 7–12 m/min, 30–40 min/day, 5 days/week, 8 weeks.	Attenuate myocardial fibrosis by inhibiting the inflammatory response.	(62)
	HFD-induced rats	Treadmill exercise: 5–10 m/min, 60 min/day, 7 days/week, 12 weeks	Alleviate myocardial fibrosis by inhibiting inflammation and apoptosis.	(63)
	Ovariectomize-induced-female spontaneously hypertensive rats	Treadmill exercise: 18–27 m/min, 60 min/day, 5 days/week, 8 weeks	Attenuate myocardial fibrosis by inhibiting the inflammatory response.	(64)
	TAC-induced heart failure mice	Swimming: 10–90 min/day, twice a day with a 6-hour interval, 3 weeks	Improve myocardial fibrosis by inhibiting oxidative stress.	(68)
	myocardial infarction mice	Treadmill exercise: 10–16 m/min, 30-60 min/day, 7 days/week, 4 weeks	Attenuate myocardial fibrosis by improving mitochondrial integrity and biogenesis and inhibiting oxidative stress	(69)
	myocardial infarction mice	Swimming: 15 min/day, once a day, 5 days/week, 8 weeks	Attenuate myocardial fibrosis by improving mitochondrial quality control and decreasing apoptosis and oxidative stress	(70)
	TAC-induced heart failure mice	Ladder-climbing exercise: 40-60% maximum load, 5 days/week, 8 weeks Moderate-intensity continuous training combined with resistance training: 65%–70% Vpeak, 60 min/time and resistance training on alternate days, 5 days/wk, 8 wk	Improved cardiac function, myocardial morphology and fibrosis by enhancing myocardial mitophagic activity.	(19)
	myocardial infarction mice	Ladder-climbing exercise: 10-75% body weight carrying load, 3 climbs/set, 8 sets/day, 5 days/week, 4 weeks	Reduce the levels of oxidative stress, apoptosis, and myocardial fibrosis and promote cardiac function.	(17)
	myocardial infarction rats	Treadmill exercise: 10-16 m/min, 10-40 min/day, 5 days/week, 10 weeks	Improve myocardial fibrosis by inhibiting inflammation and improving ECM remodeling.	(85)
	myocardial infarction mice	Treadmill exercise: 12 m/min, 60 min/day, 5 days/week, 4 weeks Ladder-climbing exercise: 75% maximum carrying load, 1 times/set, 9 sets/day, 4 weeks	Alleviate myocardial fibrosis, oxidative stress and apoptosis by increasing FGF21 protein expression	(86)
	db/db mice	Treadmill exercise: 7 m/min, 300 m/day, 5 days/week, 8 weeks	Alleviate myocardial fibrosis by inhibiting the expression of MMP9	(88)
	31-months aged rats	Treadmill exercise: 0°-12° incline up, 10.5 m/min, 10-45 min/day, 5 days/week, 12 weeks	Attenuate myocardial fibrosis and MMPs dysregulation by uppressing elevation of TIMP-1 and TGF-β.	(89)
	Male wistar rats	Treadmill exercise: 60 cm/s, 60 min/day, 5 days/week, 16 weeks	promote adverse remodeling and produce a substrate for cardiac arrhythmias.	(90)
	Male wistar rats	Treadmill exercise: 60 cm/s, 60min/day, 5 days/week, 16 weeks	Induce disproportionate RV dilatation, decreased contractility, and impaired diastolic function.	(91)
Lung	Bleomycin-induced pulmonary fibrosis mice	Treadmill exercise: 60% of the maximum speed, 60 min/day, 5 days/week, 4 weeks	Attenuate lung fibrosis by inhibiting the inflammatory response.	(97)
	Bleomycin-induced pulmonary fibrosis mice	Treadmill exercise: 60% of the maximum speed, 60 min/day, 5 days/week, 4 weeks	Attenuate lung fibrosis by inhibiting the inflammatory response.	(98)
	HFD-induced mice	Treadmill exercise: 18 m/min, 60 min/day, 7 days/week, 8 weeks	Alleviate pulmonary fibrosis by improving insulin resistance and inhibiting the inflammatory response.	(99)
	LPS-induced-COPD mice	Treadmill exercise: 11.8 m/s, 1 h/day, 5 days/week, 4 weeks	Improve pulmonary fibrosis by alleviating the inflammatory response, oxidative stress injury, and apoptosis.	(100)
	Silica suspension- induced silicosis mice	Treadmill exercise: 60% of the maximum speed, 60 min/day, 5 days/week, 4 weeks	Alleviate pulmonary fibrosis by inhibiting the inflammatory response.	(18)
	Paraquat-inducedPF mice	Wheel-running exercise: 1 h/day, 21days	Alleviate pulmonary fibrosis by inhibiting the inflammatory response and oxidative stress.	(101)
	Bleomycin-induced pulmonary fibrosis mice	Treadmill exercise: 12 m/min, 45 min/day, 5/7 days/week, 4 weeks	Improve pulmonary fibrosis by attenuating the EMT.	(104)
	Ovariectomy-induced lung injury rats	Swimming:60min/day, 7 days/week, 8 weeks	Alleviate pulmonary fibrosis by inhibiting apoptosis.	(106)
	HFD-and-STZ-induced T2DM rats	Treadmill exercise (HIIT): 80-100% Vo2max+50% Vo2max(inactive rest time), 12-30 min/time, 5 times/week, 8 weeks	Alleviate pulmonary fibrosis by inhibiting the inflammatory response, apoptosis and oxidative stress.	(107)
	PM-exposure-induced lung injury mice	Treadmill exercise: 20 m/min, 5° incline up, 60min/day 7 days/week, 1 week	Attenuate pulmonary fibrosis by inhibiting oxidative stress, pro-inflammatory responses, and apoptosis.	(109)
	Bleomycin-induced pulmonary fibrosis mice	Swimming: 1h/day, 5 days/week, 4 weeks	Attenuate pulmonary fibrosis by	(110)
	Bleomycin-induced pulmonary fibrosis mice	Treadmill exercise: 60% of the maximum speed, 60 min/session, 5 sessions/week, 4 weeks	Alleviate pulmonary fibrosis by inhibiting the inflammatory response.	(111)
Kidney	Spontaneously hypertensive rats	Treadmill exercise: 18-27 m/min, 60min/day 5 days/week, 12 weeks	Attenuate renal fibrosis by inhibiting the inflammatory response.	(117)
	db/db mice	Treadmill exercise: 5.6 m/min, 60min/day, 5 days/week, 8 weeks	Alleviate renal fibrosis by inhibiting the inflammatory response and oxidative stress.	(118)
	5/6 nephrectomy-induced CKD rats	Ladder-climbing exercise: 70% maximum carrying load, 12min/time, 8 times, 3 days/week, 10 weeks	Alleviate renal fibrosis by inhibiting the inflammatory response.	(119)
	5/6 nephrectomy-induced CKD rats	Treadmill exercise: 20 m/min, 60min/day, 5 days/week, 12 weeks	Attenuate renal dysfunction by inhibiting oxidative stress.	(120)
	High-salt-diet-induced rats	Treadmill exercise: 16-20 m/min, 60min/day, 5 days/week, 8 weeks	Attenuate renal dysfunction by inhibiting oxidative stress.	(124)
	21 months aged rats	Swimming: 30-60 min/days, 6 days/week, 12 weeks	Alleviate renal fibrosis by inhibiting the inflammatory response and oxidative stress.	(127)
	HFD-induced mice	Treadmill exercise: 70% of the maximum speed, 10-90 min/day, 5 days/week, 8 weeks	Improve of obesity-related glomerulopathy, tubulo-interstitial fibrosis, inflammation and oxidative stress.	(15)
	STZ-induced T1DM mice	Treadmill exercise: 12 m/min, 60min/day, 6 days/week, 7 weeks	Alleviate renal injury by improving mitochondrial function.	(132)
	HFD-induced T2DM mice	Treadmill exercise: 0.8 km/h, −9° tilt angle, 30-50min/day, 6 days/week, 8 weeks	Improve renal interstitial fibrosis.	(133)
	Wistar fatty (fa/fa) rats	Treadmill exercise: 13 m/min, 30min/day, 5 days/week, 8 weeks	Improve DKD by inhibiting tubulointerstitial fibrosis, inflammation, and oxidative stress.	(134)
	19 months mice	Rotary-bar training: 5 days/week, 15-60min/session time,16-24rotary/min, 48-288m/sessions, 6 weeks	Delay renal fibrosis by nducing the activation of autophagy	(136)
	STZ-induced Db mice	Treadmill exercise: 12 m/min, 45min/day, 5 days/week, 5 weeks	Improve diabetic nephropathy by inhibiting apoptosis	(137)
	Spontaneously hypertensive rats	Swimming: 10-60min/days, 6days/week,9 weeks	Attenuate renal fibrosis by inhibiting apoptosis.	(138)
	5/6 nephrectomy-induced CKD rats	Treadmill exercise: 20 m/min, 60 min/day, 5 days/week, 12weeks	Attenuate the progression of glomerular sclerosis and renal interstitial fibrosis by regulating renin-angiotensin system.	(139)
	Aldosterone-induced mice	Wheel-running exercise: 8 weeks	Attenuate the progression of glomerular sclerosis and renal interstitial fibrosis.	(140)
	19 months aged mice	Treadmill exercise: 14 m/min, 6% grade, 41 min/day, 5 days/week, 6 weeks12weeks	Attenuate renal fibrosis.	(141)
	HFD-induced T2DM mice	Treadmill exercise: (HIIT):16-26m/min+8m/m(inactive rest time) 40 min/time, 1 training/day, 10 cycles/training with 15° slope, 5 days/week, 8weeks	Induced renal injury and fibrosis.	(142)
	Spontaneously hypertensive rats	Treadmill exercise: 26m/min, 60 min/day, 5 days/week, 14 weeks	cause renal damage and fibrosis.	(143)
	Wistar rats	Resistance training: climb the ladder with a load on the tail, 55%-85%weight, 10-12 sets/time, 3-4 times/week, 12 weeks	compromise renal morphology.	(144)
Liver	HFD-induced mice	Treadmill exercise: 15-20m/min, 60min/day, 5 days/week, 16 weeks	Reduce hepatic inflammation, injury, and fibrosis by suppressing macrophage infiltration.	(154)
	D‐Galactose- induced aged rats	Treadmill exercise:45%/55% of the maximum speed, 30min/time, 4 times/week, 4 weeks	Alleviate hepatic inflammation, and fibrosis by regulating the polarization of macrophages.	(155)
	HFD-induced NAFLD mice	Treadmill exercise: 12-20m/min, 20-50min/day, 5 days/week, 8 weeks	Alleviate NAFLD by inhibiting inflammation.	(156)
	high-fat, high-carbohydrate diet -induced NASH rats	Treadmill exercise: HIIT:18-25m/min 60min/time, 3 days/week, 14weeks MIT:match the distance ran by the HIIT, 3 days/week, 14weeks	Ameliorated the progression of hepatic TG levels, inflammation, and fibrosis by reducing the infiltration of inflammatory cells.	(157)
	HFD-induced NAFLD mice	Swimming: 10-45min/days, 5days/week, 9 weeks	Ameliorate liver injury, steatosis, and fibrosis.	(160)
	High-sucrose diet-induced mice	Treadmill exercise: 15-20m/min, 15-30min/day, 12 weeks	Prevent fatty liver via improvement of hepatic lipid metabolism	(161)
	NASH-induced diet mice	Treadmill exercise: 14-20m/min, 60 min/day, 6 days/week, 8 weeks	Alleviate NASH by inhibiting lipogenesis, hepatocytes steatosis, apoptosis, insulin resistance and fibrosis.	(16)
	STZ-induced T1DM rats	Treadmill exercise: 17 m/min, 10-30 min/day, 4 weeks	Reduce liver fibrosis levels by suppressing ER stress markers and apoptosis, and improving lipid metabolism	(163)
	high-fat, high-carbohydrate diet -induced NASH rats	Treadmill exercise (HIIT and MTT): 60min/tine, 3 times/week, 14 weeks	Reduce liver weight, steatosis, inflammation, lipid accumulation, collagen deposition,and cholesterol content by inhibiting cell apoptosis	(168)
	western diet-induced NASH rats	Treadmill exercise: 20 m/min, 15% grade, 60 min/day, 5 days/week, 12 weeks	Reduce liver fibrosis via altering HSCs activation and ECM remodelling	(171)
	high-fat, high-carbohydrate diet -induced T2DM mice	Treadmill exercise: 80% of the maximal speed, 30 min/time, 3 times/week, 12 weeks	Improve liver fibrosis via decreasing HSC activation and increaseding antioxidant activity.	(172)
	Ketogenic diet and HFD -induced T2DM mice	Treadmill exercise (HIIT): 15-22m/min+8 m/min, 25% grade 40 min/time, 3 times/week, 8 weeks	Attenuates liver fibrosis by inhibiting HSC's activation and reducing protein expression of MMPS and MTIPs.	(14)
	CCl4/TAA-induced rats	Treadmill exercise: 15 m/min, 60 min/day, 5 days/week, 8 weeks	Promote HSC activation and exhibite a profibrogenic effect in the liver blunting cirrhosis regression	(174)
Skeletal muscle	HFD-induced mice	Treadmill exercise: 12–23 m/min, 25-45 min/day, 3 days/week, 22 weeks	Reduce muscle fibrosis and improve content of muscle stem/progenitor cells by reducing inflammatory signaling	(189)
	HFD-induced mice	Treadmill exercise: 16–22 m/min, 60 min/day, 3 days/week, 6 weeks	Reduce inflammation and muscle macrophage infiltration	(192)
	HFD-induced mice	Treadmill exercise: 12m/min, 5% grade, 40 min/day, 5 days/week, 12 weeks	Attenuate muscle fibrosis by reducing macrophage infiltration and inflammatory response	(193)
	db/db mice	Treadmill exercise: 10-16m/min, 5-10% grade, 10 min/day, 5 days/week, 6 weeks	Increase muscle fiber area and improve muscle fibrosis by upregulating metabolic pathways and decreasing oxidative stress.	(198)
	DMD ^mdx^ mice	Downhill running: 5 m/min-maximum speed, −15° incline, 60 min/day, 5 days/week, 7 weeks	Exacerbates endomysial fibrosis by inducing oxidative stress and oxidative DNA damage.	(200)
	MetS rats	Treadmill exercise: 15m/min, 60 min/day, 5 days/week, 4 weeks	Mitigated gastrocnemius muscle fiber atrophy and fibrosis by enhancing oxidative metabolism	(203)
	20 months aged mice	Treadmill exercise: 60% of maximum running speed, 42 min/time, 1 time/2 days, 5 months	Enhance skeletal muscle regeneration and preventing fibrosis by inhibiting CCN2 secretion from senescent MuSCs.	(22)
	38 weeks aged mice	Resistance training: climbe the ladder with a load on the tail, 6–8 sets/time, 3 times/week, 12 weeks	Prevent muscle fibrosis and atrophy via enhancing myogenic differentiation of MuSCs.	(23)
	CIM mice	Treadmill exercise: 17 m/min,−20° downhill, 30 min/day 2 weeks	Enhance skeletal muscle regeneration and inhibiting skeletal muscle fibrosis by promoting FAPs senescence.	(208)
	TA-induced mice, DNTT-induced mice	Treadmill exercise: 11 m/min, 15% grade, 60 min/day, 5 days/week, 6 weeks	Attenuate collagen deposition and adipose formation in muscles by inhibiting FAPs proliferation and promoting apoptosis in FAPs.	(209)
	Posterior transverse incision--induced rats	Stretching: 5weeks	Induce a decrease in muscle fibrosis and an increase in myogenesis in injured muscles.	(210)
	Denervation-induced rats	Stretching, 0.5, 3, or 12 cycles/min, 20 min/day, 2weeks	Suppress processes of muscle fibrosis	(211)
	Denervation-induced rats	Stretching: 7 or 15 days	Increases muscle fibrosis	(212)

## 4 Regulation of pulmonary fibrosis by exercise

### 4.1 Exercise and pulmonary fibrosis

It is widely acknowledged that exercise is a safe and effective multi-system intervention, with evidence demonstrating its efficacy in the prevention, treatment, and rehabilitation of pulmonary fibrosis, particularly in the case of IPF. Dowman et al. (92) demonstrated that eight weeks of exercise training resulted in a significant improvement in 6-minute walking distance (6MWD), dyspnea, and health-related quality of life were improved in patients with interstitial lung diseases (ILDs) in a clinically randomized controlled trial with ILD. Similarly, another study demonstrated that 12 weeks of exercise training led to significant improvements in exercise capacity, lung function, and cardiovascular function indices in patients with IPF (93). A meta-analysis demonstrated that exercise training was an efficacious intervention for enhancing cardiorespiratory endurance and quality of life in elderly patients with pulmonary fibrosis, as well as for improving the percentage of the predicted value of exertional lung capacity (94). These results indicate that exercise training may confer multifaceted clinical benefits and should be considered as a standard approach in the comprehensive treatment of IPF. In addition to aerobic exercise alone, an integrated exercise program combining respiratory training, resistance and flexibility is more effective in improving 6MWD, dyspnea levels and quality of life in patients with IPF (94, 95) (**Table 1**).

### 4.2 Mechanisms of exercise modulation of pulmonary fibrosis

#### 4.2.1 Inflammatory response

In the initial stages of pulmonary fibrosis, inflammation plays a pivotal role. The inflammatory response initiated by injury ultimately results in the onset and progression of fibrosis by recruiting neutrophils, macrophages, and lymphocytes to release a range of pro-inflammatory factors (e.g., IL-6, TNF-α), stimulate fibroblast activation, and promote collagen deposition (96). It has been demonstrated that aerobic exercise inhibits the infiltration of inflammatory cells and the expression of pro-inflammatory factors in lung tissues and bronchioles. Furthermore, it has been shown to attenuate bleomycin-induced inflammation and fibrosis in the lungs (97, 98). Eight weeks of low-intensity aerobic exercise has been found to improve pulmonary fibrosis in mice fed a high-fat diet by attenuating the chronic low-grade inflammatory response, oxidative stress injury, and activating SIRT1, among other pathways (99). Furthermore, aerobic exercise has been demonstrated to enhance the efficacy of treatments for emphysema and pulmonary fibrosis in mice with chronic obstructive pulmonary disease (COPD) by employing analogous mechanisms (100). Jin et al. (18) observed that exercise training augmented collagen deposition in the lungs of silica-exposed mice. The proposed mechanism for the enhancement of lung function, preservation of lung elastic fibers and slowing of the development of pulmonary fibrosis in silica-exposed mice is the inhibition of the macrophage-derived IL-17A–Chemokines C-X-C motif ligand 5 (CXCL5)–Chemokines C-X-C motif receptor 2 (CXCR2) inflammatory axis by exercise training (18). In a paraquat-induced lung injury model in mice, autonomous wheeled exercise has been demonstrated to retard the progression of pulmonary fibrosis, as evidenced by a reduction in the inflammatory infiltrate and a decrease in the levels of pro-inflammatory cytokines CXCL1, IL-6, TNF-α and Interferon-γ(IFN-γ) in serum and bronchoalveolar lavage fluid (BALF) in mice (101).

#### 4.2.2 Oxidative stress

Oxidative stress, defined as a state caused by the overproduction of ROS or insufficient antioxidant defenses, plays an important role in the development of pulmonary fibrosis (102). It has been demonstrated that autonomous wheeled exercise exerts a preventive effect on paraquat-induced oxidative stress. This is achieved by reducing the activities of malondialdehyde (MDA) and catalase (CAT) in lung tissues, increasing the activities of the antioxidant enzymes SOD, glutathione (GSH), and oxidized glutathione (GSSG), blocking lipid peroxidation, and activating the Nrf2 antioxidant signaling pathway (101). Furthermore, individualized exercise training was observed to significantly enhance exercise endurance and systemic antioxidant capacity in IPF patients. This ameliorative effect was found to be mediated by an increase in serum concentrations of antioxidants, including total free thiols and total glutathione, and a reduction in the production of lipid peroxidation products by exercise (103).

#### 4.2.3 Epithelial-mesenchymal transition

Epithelial-to-mesenchymal transition (EMT) is a dynamic and reversible process. In PF, alveolar epithelial cells transform into myofibroblasts via EMT, thereby directly contributing to the formation of fibrotic lesions (99). Autonomous wheeled exercise has been demonstrated to upregulate the expression of the alveolar epithelial cell marker E-cadherin, while concurrently reducing the expression of the mesenchymal cell markers α-SMA and Vimentin (101). TGF-β is widely regarded as a principal inducer of EMT, and subsequent a study has indicated that autonomous wheeled exercise inhibits the aberrant activation of the classical EMT signaling pathway, Wnt/β-catenin. By decreasing aberrant activation of the Wnt/β-catenin signaling pathway, which in turn blocks EMT and alleviates pulmonary fibrosis in alveolar epithelial cells (101). Du et al. (104) further found that exercise training attenuates EMT and pulmonary fibrosis in bleomycin-treated mice by promoting hydrogen sulfide (H2S) production and inhibiting the TGF-β1/Smad and low-density lipoprotein receptor-related proteins (LRP-6)/β-catenin signaling pathways.

#### 4.2.4 Apoptosis of epithelial cells

It has been demonstrated that the excessive apoptosis of alveolar epithelial cells plays a pivotal role in the tissue remodeling associated with pulmonary fibrosis. It has been demonstrated that bleomycin can induce a considerable increase in apoptosis-associated mediators in alveolar epithelial cells, thereby promoting the progression of pulmonary fibrosis (105). Daghigh et al. (106) employed de-ovulated rats for eight weeks of swimming. The levels of fibrosis and apoptosis markers, caspase-3, were significantly decreased in the lung tissues of de-ovulated rats, confirming that exercise may have a therapeutic potential in experimental estrogen deficiency-induced lung injury. The application of HIIT has been demonstrated to reduce the levels of apoptotic markers Bcl2-associated X protein (BAX) and BAX/B cell lymphoma/leukemia-2 (Bcl2) in BALF of mice with type 2 diabetes mellitus (T2DM). Furthermore, HIIT has been observed to improve apoptosis and lung fibrosis, which provides a partial explanation for the effects of HIIT on diabetic lung injury (107).

#### 4.2.5 Autophagy

Autophagy represents a pivotal mechanism for maintaining cellular homeostasis, and its role in pulmonary fibrosis is multifaceted. Normal levels of autophagy assist in the removal of damaged organelles and the reduction of oxidative stress and inflammation. However, excessive autophagy has been observed to exacerbate the pathological process of pulmonary fibrosis (108). A study utilizing A549 human lung epithelial cells revealed that particulate matter (PM) exposure markedly elevated ROS production, diminished cell viability, and compromised mitochondrial function (109). These findings were further validated *in vivo*, where PM-exposed mouse lung tissues exhibited significantly elevated levels of the mitochondrial fission protein dynamin-related protein 1(Drp1), enhanced mitochondrial autophagy, and induced apoptosis and PF. However, aerobic exercise was observed to significantly mitigate these adverse effects. The study demonstrated that aerobic exercise was effective in mitigating the adverse effects of PM exposure on mitochondrial autophagy. This was achieved by upregulating the expression of the anti-mitochondrial splitter protein P-Drp1 Ser637, which plays a crucial role in maintaining mitochondrial functional integrity and inhibiting excessive autophagy (109).

**Figure 2:**
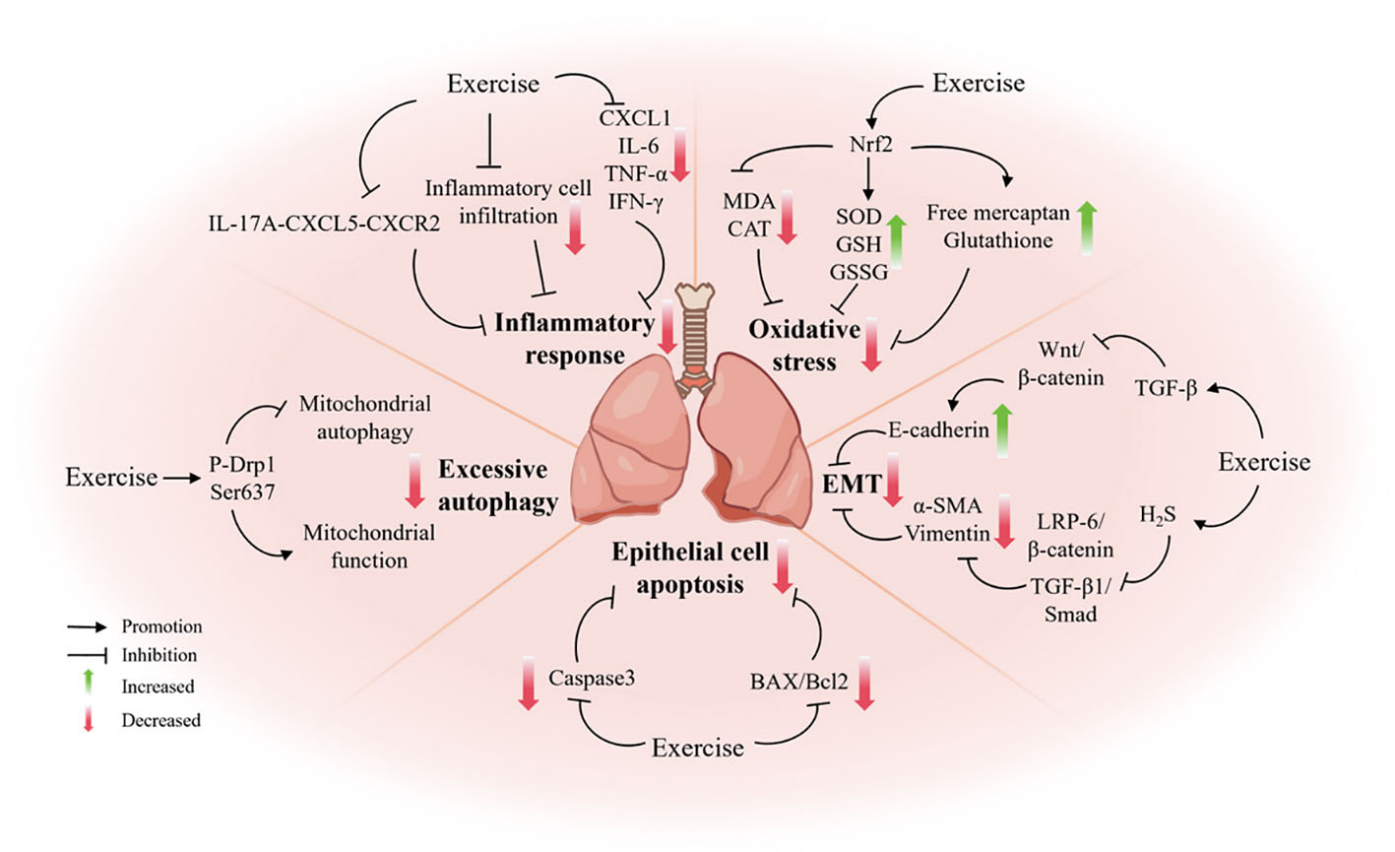
The mechanism by which exercise protects against pulmonary fibrosis.

Pulmonary fibrosis, a serious disease with the potential to be a direct threat to human life, has historically been a significant focus within the medical community. For patients with severe end-stage disease, lung transplantation remains the sole available treatment option. Despite a substantial body of evidence attesting to the substantial benefits of exercise on lung function, the precise mechanisms and specific effects on lung tissue remain unclear. Moreover, conflicting experimental results have been reported. For instance, Prata and colleagues (110) reported that exercise training had a beneficial effect on bleomycin-induced lung injury and endurance decline in mice, and delayed the progression of pulmonary fibrosis. However, El-Mafarjeh et al. (111) obtained different experimental conclusions, whereby aerobic exercise training reduced bleomycin-induced pulmonary fibrosis in a model of pulmonary fibrosis in mice. Nevertheless, this training regimen failed to inhibit pulmonary fibrosis and mechanical injury (111). Consequently, further studies and more favorable evidence are required to further investigate and clarify the effects and mechanisms of physical activity on pulmonary fibrosis (**Figure 2**) (**Table 2**).

## 5 Regulation of renal fibrosis by exercise

### 5.1 Exercise and renal fibrosis

More and more evidences show that physical exercise plays a significant regulatory role in the process of renal fibrosis. A randomized controlled trial has demonstrated that lifestyle interventions, including regular physical activity and reduced calorie intake, may delay the onset of renal impairment in patients with diabetic nephropathy. This is achieved by improving metabolic health and inhibiting oxidative stress, inflammation, and renal fibrosis (112). It has been demonstrated that swimming training can improve CKD-induced renal fibrosis by inhibiting the transdifferentiation of glomerular mesangial cell myofibroblasts (113). Furthermore, platform running has been demonstrated to improve renal fibrosis in CKD rats (114). The advantages of physical activity on chronic inflammation, cardiorespiratory fitness, muscle and bone strength, and metabolic status may elucidate the potential biological mechanisms underlying the improvement of renal function in adults with CKD, renal failure, or renal transplantation (115) (**Table 1**).

### 5.2 Mechanisms of exercise modulation of renal fibrosis

#### 5.2.1 Inflammatory response

An inflammatory response represents a direct stimulus to renal tissues. Prolonged, chronic inflammation has the potential to trigger excessive activation of immune cells and sustained release of pro-inflammatory factors. This inflammatory microenvironment promotes the expression of fibrogenic factors (e.g. TGF-β), thereby exacerbating the process of tubular epithelial cell injury and fibrosis, which is a common cause of CKD and renal fibrosis (116). The benefits of aerobic exercise in ameliorating renal inflammation and fibrosis have been widely demonstrated. Moderate-intensity treadmill training has been demonstrated to attenuate the renal inflammatory response and ameliorate renal fibrosis and kidney injury in spontaneously hypertensive rats. This is achieved by inhibiting the activation of the Toll-like receptors 4(TLR4)/NF-κB pathway and NOD-like receptor family caspase recruitment domain containing 4 (NLRC4) inflammatory bodies. Similarly, Huang et al. (117) demonstrated that 12 weeks of aerobic exercise effectively inhibited inflammatory and fibrotic pathways in the renal cortex of spontaneously hypertensive rats. This was evidenced by a significant reduction in the levels of inflammation-related proteins, such as IL-6 and cyclooxygenase-2 (COX-2), as well as a decrease in the expression of fibrosis-related proteins, including TGF-β, p-Smad2/3, CTGF, MMP-9, and MMP-2 was all reduced (117). Furthermore, eight weeks of aerobic exercise was observed to significantly ameliorate oxidative stress, inflammation and fibrosis by inhibiting the NADPH oxidase 4 (Nox4)/ROS/NF-κB/NLRP3 signaling pathway, thereby effectively alleviating kidney injury in db/db mice (118). Additionally, resistance training has been demonstrated to be an effective strategy for controlling renal fibrosis progression and inflammatory response in CKD rats, and it is also beneficial for improving muscle strength (119).

#### 5.2.2 Oxidative stress

From the onset of early CKD, the oxidative stress state is progressively enhanced, characterized by increased ROS production and decreased activity of antioxidant enzymes. This promotes tubular, endothelial cell, fibroblast damage, and fibrosis (120). The main sources of ROS production include NOX and xanthine oxidase (XO) (121). The expression of renal NOX subunits was found to be significantly increased in a rat model of chronic renal failure induced by 5/6 nephrectomy (122). In contrast, treadmill training has been demonstrated to reduce NOX and XO activities, ameliorate oxidative stress, and alleviate hypertension and renal impairment in CRF rats (123). Similarly, in a rat model of salt-sensitive hypertension, eight weeks of treadmill training significantly reduced the activity of XO, thereby alleviating oxidative stress in the kidney, but had no significant effect on NOX activity (124). Furthermore, treadmill training has been demonstrated to enhance urinary excretion of 20-hydroxyeicosatetraenoic acid and increase renal cytochrome P450 4A protein expression, which collectively facilitates the improvement of urinary protein, albumin, L-type fatty acid-binding protein excretion and glomerulosclerosis induced by a high-salt diet (124). Nitric oxide (NO) is an important antioxidant molecule that plays a pivotal role in maintaining the normal function of renal tubules, glomeruli, and tubulointerstitium. A reduction in the bioavailability of NO results in increased oxidative stress, which in turn leads to further damage to tubular epithelial and endothelial cells and an acceleration in the onset of interstitial fibrosis. Phosphorylation of endothelial nitric oxide synthase (eNOS) represents a pivotal step in the synthesis of NO (125). It has been demonstrated that rats fed a high fructose diet exhibit enhanced XO and NOX activity, while renal eNOS phosphorylation and NO bioavailability are inhibited. Long-term exercise interventions have been demonstrated to reverse these changes, exerting antihypertensive and renoprotective effects (126). Furthermore, swimming exercise has been shown to reduce MDA levels in the kidney and increase MnSOD activity, thereby reducing oxidative stress in aged rats (127). These findings suggest that the inhibition of oxidative stress may be a key mechanism by which exercise training exerts a renoprotective effect.

#### 5.2.3 Lipid metabolism

Recent evidence has demonstrated that disorders of lipid metabolism and aberrant lipid deposition in renal tissues are significant characteristics of CKD. This is not only a defining feature of the pathological process but may also serve as a crucial driver of renal fibrosis. Disturbed renal lipid metabolism has been demonstrated to increase lipotoxicity, trigger oxidative stress and inflammatory responses, and activate pro-fibrotic signaling pathways (128). It is demonstrated that exercise may ameliorate renal fibrosis by enhancing fatty acid oxidation and reducing lipid deposition and lipotoxicity. It has been demonstrated that delayed endurance exercise training can reduce renal ectopic lipid deposition in obese mice (15). Furthermore, exercise training has been demonstrated to alleviate obesity-induced CKD by upregulating AMPK activity, enhancing Acetyl-CoA Carboxylase (ACC) phosphorylation and promoting increased fatty acid oxidation in the kidneys of obese mice (15). Peroxisome proliferator-activated receptor alpha (PPARα) plays a pivotal role in the regulation of renal lipid metabolism. It protects the kidney from lipid accumulation and lipotoxicity by promoting fatty acid uptake, catabolism, and transport to maintain renal homeostasis (129). The activation of PPARα in the kidneys of aged rats by swimming exercise resulted in the increased expression of PPARα-targeted microRNAs (miR-21 and miR-34a). This led to the attenuation of renal lipid accumulation, the suppression of renal inflammation and oxidative stress, and the reduction of renal fibrosis (127).

#### 5.2.4 Mitochondrial function

Mitochondrial dysfunction is a significant contributor to renal fibrosis, which accelerates the onset and progression of fibrosis through mechanisms such as aberrant energy metabolism, oxidative stress, mitochondrial DNA damage, accelerated EMT, and autophagy inhibition (130). SIRT1 is a NAD-dependent deacetylase that plays a pivotal role in regulating cellular metabolism and modulating mitochondrial function as a key factor in the regulation of energy metabolism. SIRT1 exerts a nephroprotective effect by mediating renal anti-fibrotic and inflammatory responses through the modulation of multiple signaling pathways and biological processes (131). Tang et al. (132) observed that T1DM mice exhibited renal tissue fibrosis accompanied by mitochondrial dysfunction (reduced mitochondrial ATP production and decreased membrane potential) and increased mitochondrial superoxide production. Conversely, aerobic exercise was found to improve mitochondrial function by up-regulating the expression of SIRT1/PGC-1α in the kidneys, which was associated with a protective effect against renal injury. In a T2DM mouse model, aerobic exercise has also been demonstrated to inhibit the TGF-β1/Smad3 pathway by upregulating SIRT1 expression, thereby alleviating renal interstitial fibrosis in T2DM mice (133).

#### 5.2.5 Autophagy

Autophagy, as an essential physiological function for cells to reduce mitochondrial damage and oxidative stress, plays a critical role. Dysregulation of this function is a key mechanism in the initiation of renal fibrosis. Studies by Juszczak et al. (15) and Monno et al. (134) have demonstrated that AMPK-mediated restoration of autophagy is an important biological mechanism of exercise training to ameliorate renal fibrosis. Specifically, in mice and rats with high-fat diets and T2DM, running training activated AMPK in the kidneys, increased the phosphorylation level of UNC-51-like kinase 1 (ULK-1) and inhibited the activity of mammalian target of rapamycin complex 1(mTORC1), respectively. This resulted in the restoration of metabolic disorder-induced inhibition of autophagic fluxes, which in turn improved renal fibrosis (15, 134). In addition to metabolic factors, natural aging has also been demonstrated to contribute to the development of renal fibrosis (135), which may also be a manifestation of the decline of organ function that accompanies the aging process. The study by Bao et al. (136) also confirmed the effect of physical exercise It has been demonstrated that aerobic exercise training may improve renal aging-induced fibrosis by modulating the TGF-β1/Transforming growth factor beta-activated kinase 1 (TAK1)/Mitogen-activated protein kinase kinase 3 (MMK3)/p38MAPK signaling pathway, thereby inducing autophagy activation, reducing extracellular matrix synthesis, delaying EMT and thus improving renal fibrosis in aged mice.

#### 5.2.6 Apoptosis

Diabetic nephropathy represents a prevalent microvascular complication of diabetes mellitus, characterized by structural and functional damage to the kidneys. Diabetic kidney injury is closely associated with an increased rate of apoptosis in renal cells. Furthermore, exercise training has been demonstrated to enhance renal SIRT1 expression in diabetic mice, while simultaneously inhibiting the p53-mediated pro-apoptotic pathway and suppressing renal fibrosis (137). In particular, the exercise of platform running was observed to upregulate the expression of cystathionine-β-synthase (CBS) and cystathionine-γ-lyase (CSE) and enhance the production of endogenous H2S in renal tissues. This resulted in the improvement of diabetic renal fibrosis through the regulation of the SIRT1/p53 apoptotic pathway, which in turn led to the amelioration of renal injury in diabetic individuals (137). Duang et al. (138) reported that eight weeks of swimming training significantly down-regulated the expression of key proteins in the TGF-β1/Smad signaling pathway in the kidneys of spontaneously hypertensive rats. These proteins included TGF-β1 and Smad2/3. The expression of the pro-apoptotic Bax protein was found to be downregulated, while the anti-apoptotic protein Bcl-2 and Smad7 were upregulated. This resulted in a reduction in renal injury in diabetic nephropathy. The expression of these proteins exerted an anti-apoptotic effect on renal cells, thereby attenuating renal interstitial fibrosis.

Furthermore, dysfunction of the renal renin-angiotensin system and senescence of renal tubular epithelial cells have been identified as additional factors. Furthermore, it represents a crucial mechanism in the pathogenesis of renal fibrosis. Conversely, exercise training has been demonstrated to exert a beneficial effect on the progression of glomerulosclerosis and interstitial fibrosis in rats with chronic renal failure by modulating the function of the renal renin-angiotensin system (139, 140). A recent study demonstrated that 12 weeks of aerobic exercise was able to ameliorate renal fibrosis associated with aging by inhibiting the TGF-β1/p53/miR-34a signaling pathway, upregulating the expression of the anti-aging protein Klotho, and further inhibiting its downstream TGF-β1/Smad3 and β-linker signaling pathways (141).

**Figure 3:**
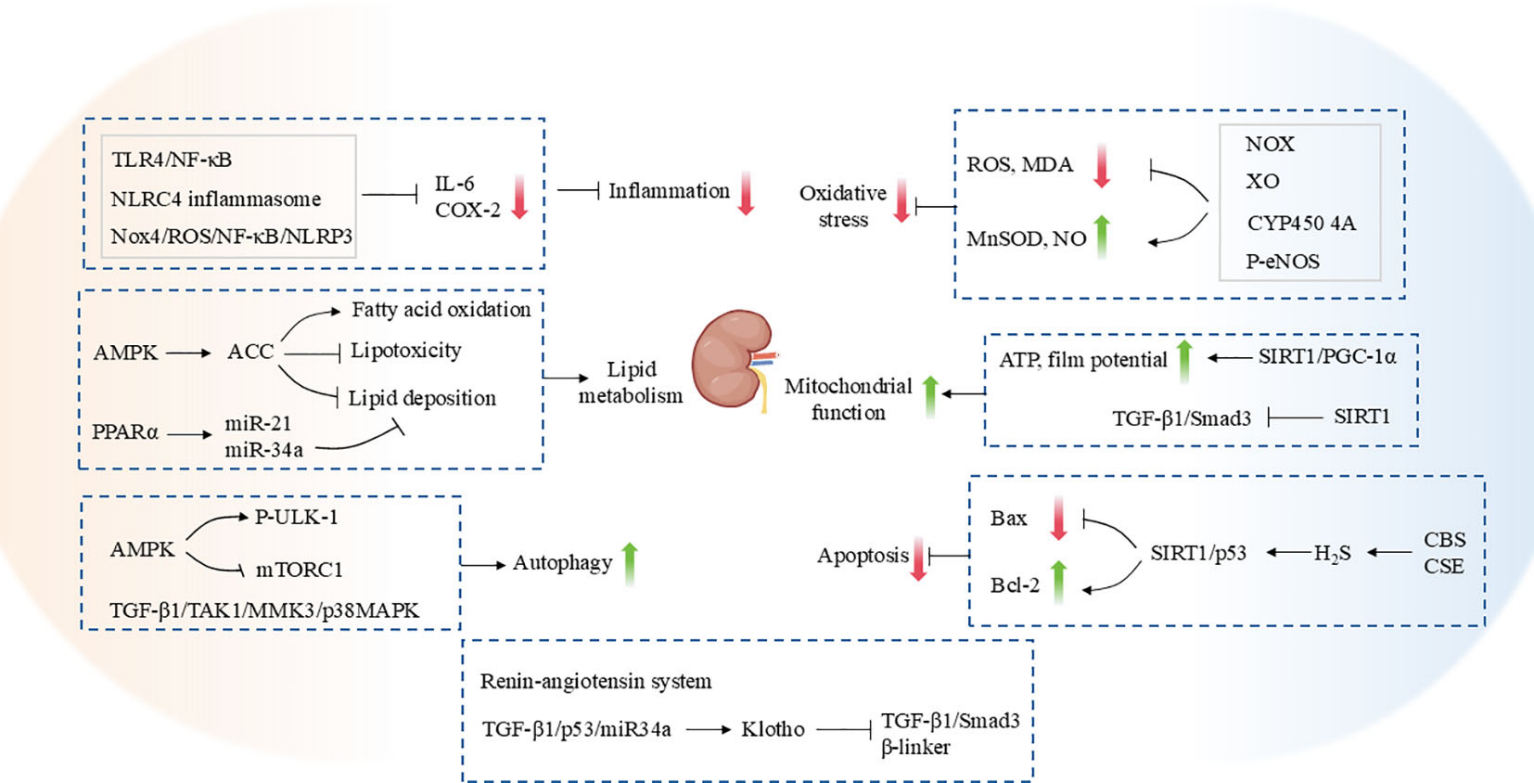
The mechanism by which exercise protects against renal fibrosis.

While the aforementioned studies have corroborated the substantial beneficial effects of exercise on renal function and renal fibrosis, it is imperative to exercise caution when selecting the appropriate exercise intensity. It is possible that exercising at an intensity that is too high may not only fail to achieve the expected protective effect but may even trigger potential damage to renal tissues and aggravate pathological changes. Zheng et al. (142) found that HIIT increased the accumulation of serum creatinine and glycogen in the kidneys of T2DM mice, up-regulated the fibrosis-related proteins TGF-β1, CTGF, COL3A1, α-SMA expression, which induced renal injury and fibrosis. A study was conducted to investigate the impact of varying exercise intensities on RF in spontaneously hypertensive rats (SHR). The findings revealed notable discrepancies in the effects of different exercise intensities on renal fibrosis (143). In particular, moderate-intensity exercise was observed to significantly reduce lactate levels in the kidneys and blood of SHR, while simultaneously downregulating the expression of fibrosis-related proteins Transient receptor potential cation channel subfamily V member 4 (TRPV4), TGFβ-1, p-Smad2/3, and CTGF. Conversely, high-intensity exercise has been observed to result in elevated lactate levels, which in turn has been shown to activate the TRPV4-TGFβ1-SMAD2/3-CTGF signaling pathway, thereby exacerbating renal fibrosis (143). Furthermore, Aparicio et al. (144) demonstrated that high-intensity strength training resulted in the development of renal interstitial fibrosis in rats, leading to damage to the normal morphological structure of the kidney. These results suggest that exercise intensity may be a crucial factor in regulating renal fibrosis. Consequently, physical activity may exert a bidirectional modulatory effect on renal fibrosis, depending on the type of exercise (**Figure 3**) (**Table 2**).

## 6 Regulation of liver fibrosis by exercise

### 6.1 Exercise and liver fibrosis

Exercise training has been demonstrated to be a safe and effective intervention for the prevention and treatment of liver fibrosis (145, 146). Stine et al. (147) found that moderate-intensity aerobic exercise at 30 minutes, five times a week for 20 weeks, resulted in the down-regulation of serum Alanine transaminase (ALT) expression in patients with NAFLD. This provides preliminary evidence that exercise may improve liver fibrosis in patients with NAFLD. A human study in patients with metabolic syndrome observed that 150 minutes of moderate-intensity physical activity per week for six months resulted in the suppression of the expression of Aspartate transaminase (AST), a marker of liver injury (148). A two-year clinical study observed that a combination of moderate-intensity brisk walking for 30–60 minutes, three to four times per week, and dietary intervention resulted in a reduction in NAFLD-FS scores in patients with NAFLD (149). While numerous studies have demonstrated the efficacy of exercise training in ameliorating liver fibrosis, other research has indicated that prolonged, high-intensity exercise may not be as beneficial. For instance, a 12-week program of rigorous cycling and resistance training did not lead to a notable reduction in liver fibrosis in patients with NASH (150) (**Table 1**).

### 6.2 Mechanisms of exercise modulation of liver fibrosis

#### 6.2.1 Inflammatory response

The inflammatory response plays a pivotal role in the pathogenesis of liver fibrosis, exerting a profound influence throughout the entire process, from the initial stages of fibrosis to its progression. Chronic liver injury (e.g., viral hepatitis, alcoholic liver disease, non-alcoholic fatty liver disease, etc.) can result in the activation of immune cells and the sustained release of inflammatory factors in the liver. Uncontrolled chronic inflammation over an extended period represents the primary trigger for hepatic stellate cell (HSC) activation and excessive deposition of ECM (151–153).

In light of the considerable interest in inflammatory responses in the hepatic fibrotic process, Kawanishi et al. (154) observed that exercise training reduced hepatic inflammation, injury and fibrosis in NASH mice by inhibiting macrophage infiltration. In addition to pathological factors, the process of aging increases the susceptibility of the liver to disease and causes inflammation of liver tissue, which in turn leads to liver fibrosis. Furthermore, exercise training has been demonstrated to exert a beneficial influence on age-related liver fibrosis. In a study by Wasityastuti et al. (155), low-intensity running exercise was observed to markedly attenuate the extent of liver fibrosis in D-galactose-induced senescent mice. Additionally, moderate running exercise was found to facilitate the restoration of injured liver tissues to a non-senescent state to a certain extent. It was demonstrated that eight weeks of platform running exercise promoted irisin expression, inhibited myeloid differentiation factor 2 (MD2)-TLR4 binding and suppressed the expression of downstream inflammatory factors IL-6, IL-1β, TNF, chemokine (C-C motif) ligand 2 (CCL2), intercellular adhesion molecule-1 (ICAM1), and vascular cell adhesion molecule-1 (VCAM1) in the livers of NAFLD mice, as well as COL1A1 and TGF-β (156). Irisin was observed to elicit comparable effects to those of exercise in AML cells. This indicates that exercise inhibits the binding of the MD2-TLR4 complex by promoting irisin production, which downregulates the inflammatory response and thereby ameliorates liver fibrosis (156). Given the wide range of benefits associated with exercise, it has been demonstrated that HIIT is more effective in ameliorating hepatic steatosis, inflammation and fibrosis in NASH mice compared to moderate-intensity continuous training. This suggests that selecting an appropriate type of exercise is crucial (157).

#### 6.2.2 Lipid metabolism

The liver plays a pivotal role in lipid metabolism within the body. In disease states such as obesity, diabetes mellitus, or fatty liver, disorders of body lipid metabolism, hepatic fat deposition, lipotoxicity, and hepatic oxidative stress and chronic inflammation induced on this basis have been demonstrated to be a significant pathomechanism for the induction of liver fibrosis (158, 159). The loss of lipids is an important objective in the comprehensive treatment of metabolic diseases such as NAFLD. The weight loss effect brought about by exercise is also an important method of preventing the progression of NAFLD/NASH to liver fibrosis. Qian et al. (160) reported that swimming exercise blocked the upregulation of Hydroxymethylglutaryl CoA synthetase 2 (HMGCS2) in the livers of NAFLD mice, inhibited the activation of the Wnt3a/β-catenin pathway, improved lipid metabolism, and alleviated hepatic lipid accumulation, thereby preventing inflammation and fibrosis induced by lipotoxicity. A study observed that regular exercise suppressed the expression of heat shock protein 47 (HSP47), a marker of liver fibrosis, in mice with a high-sucrose diet-induced fatty liver. From a mechanistic perspective, regular exercise has been shown to promote the levels of carnitine palmitoyltransferase II, acyl-coenzyme A dehydrogenase, and trifunctional enzymes in the liver, thereby inhibiting liver fibrosis (161). Furthermore, another study has demonstrated the role of exercise in the amelioration of liver fibrosis. The research team observed that exercise suppressed the expression of plasma triglycerides, phosphatidic acid, and phosphatidylglycerol in high-fat diet (HFD)-induced NAFLD mice, thereby improving hepatic lipid metabolism and inhibiting pathological steatosis (162). Furthermore, it was demonstrated that the lipid metabolism genes FXR, SREBP-1c and FAS were involved in the improvement of the liver fibrosis process in T1DM rats by exercise (163). A recent study found that 8 weeks of aerobic exercise can promote the expression of Kruppel-like factor 10 (KLF10) through the cyclic adenosine monophosphate (cAMP)/protein kinase A (PKA)/cyclic AMP response element-binding protein (CREB) pathway and regulate the fumarate hydratase 1 (Fh1)/fumarate/H3K4me3 pathway to suppress the expression of fat synthesis genes fatty acid synthase (FAS), acetyl-CoA carboxylase Alpha (ACACA), and stearoyl-CoA desaturase 1 (SCD1). This inhibition reduced lipid synthesis in the liver of NASH mice, subsequently improving hepatic steatosis and fibrosis (16).

#### 6.2.3 Apoptosis

Apoptosis is a form of programmed cell death that is mediated by the Caspase family of enzymatic reactions. It is characterized by a series of morphological changes, including cellular crumpling, nuclear chromatin condensation, and the formation of apoptotic vesicles (164). The evidence indicates that hepatocyte apoptosis can lead to a pro-fibrotic response through the activation of the Fas death receptor (165, 166). A recent study observed that exercise training effectively suppressed the expression of fibrotic genes, including TGFβ and Fibronectin 1(FN1), as well as downregulated the expression of caspase3, caspase7 and caspase9 apoptotic markers in the livers of mice subjected to a HFD. The aforementioned results indicate that exercise has the potential to ameliorate liver fibrosis by inhibiting apoptosis (167). Mohammadpour-Asl et al. (163) observed that a four-week exercise intervention resulted in a significant reduction in the expression of apoptotic markers caspase 8 and caspase 12 in the liver, thereby inhibiting liver fibrosis. Studies have shown that physical activity regulates epigenetic mechanisms, which also mediate the beneficial effects of exercise on liver fibrosis. One study reported that 14 weeks of both HIIT and moderate-intensity continuous training (MIT) suppressed the expression of liver fibrosis markers COL1A1 and actin alpha 2, smooth muscle (Acta2) in NASH mice induced by a high-fat, high-carbohydrate diet, while also downregulating the expression of isopentenyl-diphosphate delta isomerase 1 (Idi1) mRNA. Notably, the effects of HIIT were more pronounced than those of MIT (168). Further research revealed that exercise training improved liver fibrosis in NASH mice by downregulating lysine methyltransferase 2D (KMT2D)-mediated IDI1 histone methylation, which inhibits apoptosis (168).

#### 6.2.4 ECM remodeling

Hepatocytes are non-parenchymal cells that are unique to the liver. In the absence of any pathological conditions, hepatic stellate cells (HSCs) are in a quiescent state and are primarily responsible for the storage of vitamin A and lipids. However, in the context of chronic liver injury induced by viral infections or metabolic diseases, HSCs undergo activation and transformation into myofibroblast-like cells. These cells secrete pro-fibrotic factors and participate in the excessive deposition of ECM, thereby driving fibrosis progression (169, 170). It has been demonstrated that the activation of quiescent HSCs and their differentiation into myofibroblasts represent pivotal events in the transition from hepatic steatosis to fibrosis (153). Numerous studies have demonstrated that exercise intervention has a significant effect in improving liver fibrosis, one of the mechanisms being the inhibition of HSC activation and regulation of ECM remodeling. Linden and colleagues (171) have reported that aerobic exercise exerts a certain degree of influence on the amelioration of liver fibrosis was observed, yet these effects did not result in a significant reduction in body weight or amelioration of hepatic steatosis. It was proposed that these improvements may be due to the inhibition of HSC activation and the regulation of ECM remodeling (171). In other similar studies, researchers also found that both aerobic training and HIIT could improve liver fibrosis in T2DM mice by inhibiting the activation of HSCs (14, 172). In addition, HSCs are one of the main sources of MMPs and TIMPs in the liver (173). The balance between MMPs and TIMPs is also crucial for maintaining the homeostasis of ECM in the liver. Exercise has been shown to regulate the activity and expression of key ECM remodeling enzymes, such as MMP-2, MMP-9, MMP-12, and TIMP-1, improving the disruption of ECM remodeling in pathological states and exerting a protective effect against liver fibrosis (14, 171). However, it is important to note that in some cases, exercise can activate HSCs and exacerbate liver fibrosis. Reports indicate that in rat models of advanced liver cirrhosis induced by carbon tetrachloride (CCl4) or thioacetamide (TAA), moderate-intensity endurance exercise promoted HSC activation, impairing the natural regression of liver fibrosis (174). Similarly, in early-stage cirrhosis and healthy rats, 8 weeks of moderate-intensity endurance exercise also exacerbated liver fibrosis (174). The accumulation of ammonia in the liver induced by exercise may, to some extent, explain the harmful effects of exercise that sustain HSC activation and fibrosis deposition (174).

**Figure 4:**
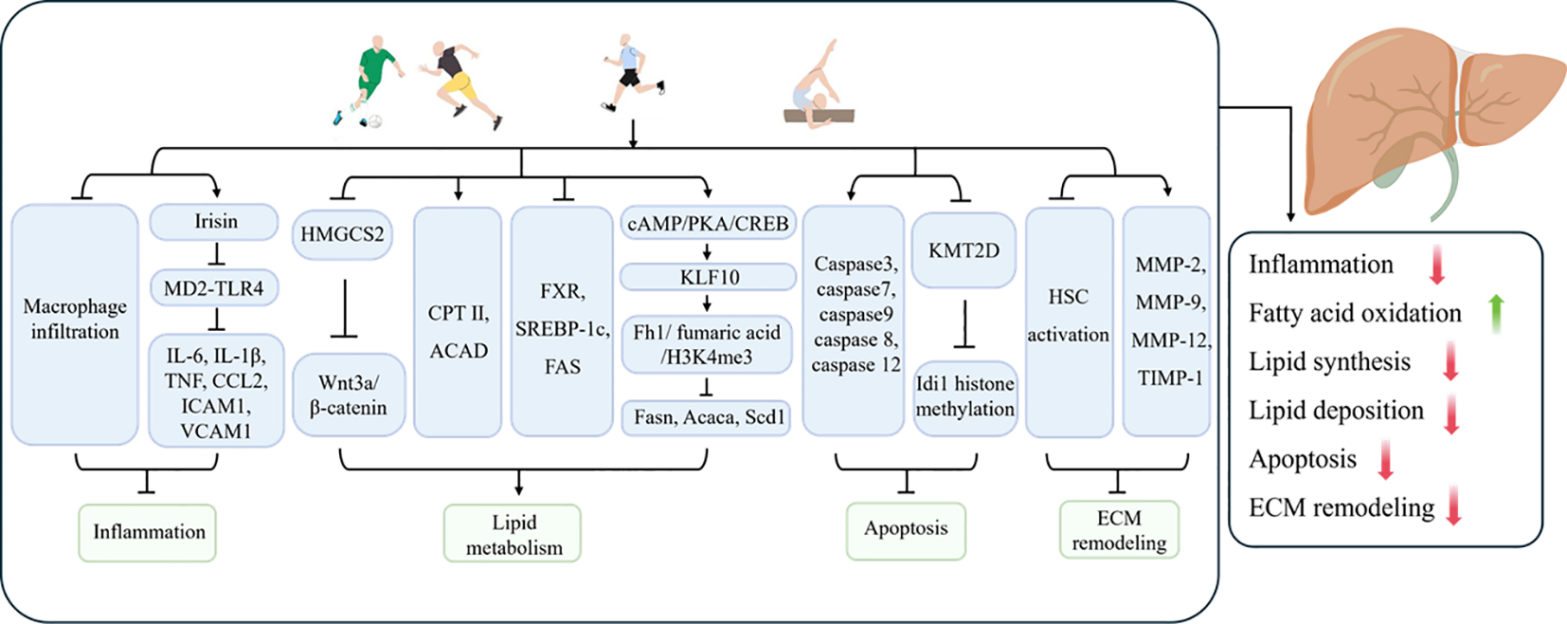
The mechanism by which exercise protects against liver fibrosis.

In conclusion, exercise has been demonstrated to exert a beneficial effect on liver fibrosis by inhibiting inflammatory responses, HSCs overactivation, and apoptosis. In addition, the improvement of liver lipid metabolism homeostasis and ECM remodeling homeostasis also mediated the effect of exercise in inhibiting liver fibrosis. Notably, the majority of the current evidence indicates that HIIT has a superior efficacy in the amelioration of liver fibrosis. However, given that the majority of studies incorporated both dietary and exercise components, and were limited by low statistical efficacy, the majority of studies examining exercise interventions for NAFLD only confirmed a reduction in intrahepatic triglyceride levels and an improvement in hepatocellular steatosis following exercise interventions. However, no improvement in established liver fibrosis was observed. A limited number of studies have demonstrated a reduction in fibrosis, such as high-intensity interval training, which has recently been identified as a novel form of exercise, with an improvement in liver stiffness (-16.8%) following a period of high-intensity interval training. However, these benefits do not appear to be associated with weight loss or fat loss (175). Consequently, further studies are required to investigate whether the fat loss effects of exercise can actually impact liver fibrosis (**Figure 4**) (**Table 2**).

## 7 Regulation of skeletal muscle fibrosis by exercise

### 7.1 Exercise and skeletal muscle Fibrosis

A study was conducted to assess the impact of resistance training on skeletal muscle in patients with idiopathic inflammatory myopathies. The researchers observed that seven weeks of resistance training significantly reduced the deposition of ECM and the content of type I collagen in skeletal muscle, accompanied by improved muscle function and the downregulation of the expression of pro-inflammatory and pro-fibrotic-related genes. These findings suggest that physical activity helps to reduce inflammation and fibrosis in skeletal muscle due to idiopathic inflammatory myopathy (176). Skeletal muscle is one of the organs most susceptible to the effects of the aging process. As a consequence of the aging process, the ECM of skeletal muscle undergoes fibrotic changes, which are accompanied by a progressive loss of muscle strength and power, a condition known as sarcopenia. The prevalence of sarcopenia in the elderly population is estimated to be between 5 and 10 percent (177). A clinical trial conducted by Gumpenbergre and colleagues (178) demonstrated that a single acute exercise stimulus markedly influenced the expression of mRNA levels of genes associated with ECM remodeling, including MMP3, MMP15, MMP9, COL1A1, and others, in the skeletal muscle of the elderly. This provides further evidence to support the efficacy of exercise in combating age-related muscle fibrosis.

### 7.2 Mechanisms of exercise modulation of skeletal muscle fibrosis

#### 7.2.1 Inflammatory response

The repair of muscle damage is dependent on the inflammatory response, which plays a key role in this process. However, when the inflammatory response persists without subsiding, chronic and non-permanent inflammation observed in skeletal muscle with idiopathic inflammatory myopathies, malnutrition, aging and metabolic disorders induces myofibroblasts to proliferate, which in turn leads to excessive extracellular matrix deposition (179–183). Furthermore, persistent inflammation can also be associated with impaired function of immune cells, microsatellite cells/muscle stem cells (MuSCs) and Fibro-adipogenic progenitor (FAP) cells in skeletal muscle, which in turn impairs regeneration and may eventually lead to fibrosis (179–183). The anti-inflammatory effects of exercise have been well documented in scientific literature. A substantial body of evidence from numerous studies has demonstrated that exercise has an anti-inflammatory effect, reducing the release of pro-inflammatory factors (e.g., TNF-α, IL-1, IL-6) and increasing the production of anti-inflammatory cytokines (e.g., IL-10). This reduces chronic inflammatory responses and attenuates muscle fibrosis (184–186). NF-κB is a pivotal nuclear transcription factor in the modulation of inflammatory and immune responses. Its activation is implicated in the pathological processes of inflammation and fibrosis in skeletal muscle (187, 188). It has been demonstrated that exercise can downregulate the phosphorylation level of NF-κB in the skeletal muscle of mice with precancerous colorectal lesions, thereby ameliorating the development of skeletal muscle fibrosis induced by HFD (189). Furthermore, the functional homeostasis of immune cells (e.g., macrophages) in skeletal muscle plays a role in the inflammatory response and tissue repair following skeletal muscle injury (190, 191). Furthermore, physical exercise has been demonstrated to regulate the homeostatic balance of macrophages in skeletal muscle. A study has confirmed that six weeks of endurance training inhibited macrophage infiltration in the skeletal muscle of obese mice and promoted macrophage polarization from a pro-inflammatory M1 phenotype to an anti-inflammatory M2 type, thereby alleviating muscle inflammation (192). It is noteworthy that the current study has revealed a dual role for M2-type macrophages in the repair of skeletal muscle injury. On the one hand, it can promote the dissipation of inflammation by secreting anti-inflammatory factors such as IL-10; on the other hand, M2-type macrophage-derived TGFβ1 is also a key factor in activating the transformation of fibroblasts into myofibroblasts and promoting skeletal muscle regeneration (190). The presence of fibrosis in skeletal muscle has been observed in previous studies. HFD has been shown to induce macrophage infiltration in skeletal muscle, resulting in an increase in the number of M2-type macrophages. Additionally, the expression of TGFβ1 protein, which activates the TGF-β/Smad signaling pathway, has been linked to the induction of skeletal muscle inflammation, collagen fiber deposition and fibrosis (193). Conversely, research indicates that platform running exercise inhibits macrophage infiltration and the secretion of pro-inflammatory factors, down-regulates TGFβ1 protein expression, and alleviates inflammation and fibrosis in skeletal muscle (193).

#### 7.2.2 Oxidative stress

It is frequently the case that chronic inflammation of tissues is associated with oxidative stress injury. The generation of a substantial quantity of ROS in the context of oxidative stress results in cellular damage, thereby intensifying the inflammatory response and precipitating tissue injury. Factors such as chronic disease, aging and ischemic injury frequently result in elevated oxidative stress levels (194). Elevated levels of oxidative stress can accelerate the progression of fibrosis by promoting damage to muscle cells and abnormal activation of fibroblasts (195–197). It was demonstrated that aerobic exercise markedly reduced oxidative stress and fibrosis in the gastrocnemius muscle of db/db mice, while enhancing the expression of antioxidant proteins, including MAPK1, SIRT2, and cellular components (CCs) (198). This indicates that aerobic exercise can mitigate diabetes-induced skeletal muscle fibrosis by regulating redox homeostasis. Irisin, a muscle-secreted exercise-inducible polypeptide hormone, has been demonstrated to mediate a range of beneficial effects associated with exercise (75). Wu et al. (199) demonstrated that irisin can mitigate the redox imbalance and alleviate the D-galactose imbalance through the activation of the PI3K/Akt/Nrf2 signaling pathway. Furthermore, it was observed that irisin can mitigate D-galactose-induced senescence of skeletal muscle fibroblasts and excessive ECM deposition in the cells. This also provides a novel perspective through which to elucidate the ameliorative effect of exercise on skeletal muscle fibrosis. Conversely, a separate study indicated that seven weeks of downhill running training resulted in heightened oxidative stress and oxidative DNA damage in the gastrocnemius muscle of Duchenne muscular dystrophy (DMD) mdx mice, leading to increased endomysial fibrosis (200). Consequently, the regulatory impact of exercise on oxidative stress may be twofold, underscoring the necessity for patients to select an appropriate exercise regimen tailored to their specific circumstances.

#### 7.2.3 Metabolic function

Skeletal muscle is one of the most active organs in energy metabolism and plays a pivotal role in the body’s glycolipid metabolism (201). Metabolic disorders such as diabetes and obesity frequently result in skeletal muscle insulin resistance, fatty acid accumulation and lipotoxicity, and oxidative stress, which in turn exacerbate skeletal muscle inflammation and fibrosis (202). Exercise training has been demonstrated to be an effective non-pharmacological intervention for the prevention and slowing of the progression of fibrosis in skeletal muscle, through the improvement of disorders of skeletal muscle glycolipid metabolism. Huang et al. (198) observed that aerobic exercise improved glycemic homeostasis, down-regulated the TGF-β pathway, and markers of fast-twitch fibrosis in db/db mice. The expression of COL1A1, COL4A2, laminin (LAMA4) and endoglin (ENG) proteins was effectively inhibited, resulting in a reduction in gastrocnemius fibrosis. The proteomic analysis revealed that aerobic exercise had a marked effect on the protein levels associated with insulin sensitivity, glucose metabolism, fatty acid metabolism, and other signaling pathways (198). This indicates that aerobic exercise may have a beneficial role in the treatment of T2DM-induced skeletal muscle fibrosis by restoring insulin sensitivity and metabolic function. Furthermore, aerobic exercise was observed to exert an ameliorative effect on STZ-induced fibrosis in the longissimus and soleus muscles of T1DM rats (13). It is proposed that the Neuregulin 1(NRG1)/Erb-b2 Receptor Tyrosine Kinase 2 (ErbB2) pathway may be downregulated as a result of exercise, which may in turn mediate the observed ameliorative effect (13). Similarly, in a rat model of metabolic syndrome (MetS), researchers observed that four weeks of aerobic treadmill training enhanced oxidative metabolism in the gastrocnemius muscle of MetS rats, as evidenced by an increase in fatty acid oxidation and oxidative phosphorylation activity. Additionally, this training regimen attenuated MetS-induced gastrocnemius muscle fiber atrophy and fibrosis (203).

#### 7.2.4 Cell regeneration and tissue repair

Adult skeletal muscle exhibits a robust regenerative capacity, with MuSCs undergoing activation and participation in the regenerative program of muscle fibers following tissue injury (204). However, the regenerative capacity of MuSCs is known to decline with age. It has been demonstrated that the activation of the Wnt/β-catenin signaling pathway in aged skeletal muscle results in a shift of MuSCs from a myofibroblast lineage to a fibroblast lineage. This impairs muscle regeneration and enhances the fibrotic response (205). Ori et al. (23) reported that 12-week resistance stair-climbing training resulted in the down-regulation of β-catenin expression in MuSCs and fibroblasts within the skeletal muscle of aged mice, as well as in fibroblasts. The enhanced expression of β-catenin promoted the differentiation of MuSCs, thereby mitigating the development of skeletal muscle atrophy and fibrosis. Conversely, the downregulation of circulating complement component 1q (C1q) levels by resistance training in the senescent state may mediate the inhibitory effect of Wnt signaling in muscle (23). The secretion of CTGF/connective tissue growth factor 2(CCN2) by aged MuSCs is increased, which in turn promotes fibroblast proliferation and impairs the regenerative capacity of MuSCs, thus facilitating the development of skeletal muscle fibrosis (22). It is noteworthy that exercise has demonstrated the capacity to mitigate this deleterious effect. Subsequent a study has revealed that sustained aerobic exercise training at moderate intensity has the effect of inhibiting CCN2 secretion from senescent MuSCs, significantly ameliorating the senescence-induced decline in locomotor activity, improving regenerative repair of skeletal muscle and preventing skeletal muscle fibrosis in senescent mice (22). FAPs are another type of highly heterogeneous multipotent stem cells in skeletal muscle, with the potential for multi-lineage differentiation including adipogenesis, fibrosis, osteogenesis, and chondrogenesis (206). Additionally, it represents a significant source of fibroblasts within skeletal muscle and plays a pivotal role in intramuscular fat deposition and fibrosis (206). An excessive accumulation of FAPs during the recovery phase following skeletal muscle injury frequently results in fibrosis and fatty infiltration of the muscle, thereby impairing muscle recovery (207). The targeted regulation of FAPs homeostasis represents a promising avenue for intervention in a range of skeletal muscle-related disorders. It has been demonstrated that exercise can facilitate skeletal muscle regeneration and reduce skeletal muscle fibrosis in mice by inducing the senescent phenotype and apoptosis of FAPs (208). Furthermore, exercise exerts an indirect regulatory influence on the fate of FAPs by inducing the secretion of skeletal muscle factors. It has been demonstrated that the secretion of Musclin by skeletal muscle during exercise inhibits the proliferation of FAPs and promotes apoptosis in FAPs by up-regulating the expression of filamin A interacting protein 1 like (FILIP1L) in FAPs. This prevents the abnormal accumulation of FAPs, which in turn inhibits collagen deposition and adipose formation in injured or wasted muscles (209).

In clinical practice, in addition to active forms of exercise such as aerobic exercise and resistance training, passive exercise is also an important component of exercise therapy. Furthermore, evidence indicates that passive exercise is an effective intervention for improving skeletal muscle fibrosis. It has been demonstrated that mechanical stretching exercises of skeletal muscle can markedly enhance the resolution of muscle fibrosis resulting from trauma (210) and denervation (211). It is important to note that further research is required to ascertain the safety and reliability of stretching exercises. This is because slower rates of stretching and intermittent stretching have been demonstrated to upregulate the expression of genes such as TGF-β1, thereby exacerbating skeletal muscle fibrosis in denervated rats (211, 212). Furthermore, exercise therapy can be combined with tissue engineering techniques, among others, to facilitate the restoration of muscle function. Endo and colleagues have developed a scaffold with layered porosity that gradually releases insulin-like growth factor 1 (IGF-1) following implantation into muscle tissue (213). In a mouse model of volumetric muscle loss (VML), the implantation of this scaffold and aerobic exercise were observed to promote skeletal muscle repair and the generation of neuromuscular junctions in newly regenerated tissues in a synergistic manner, which resulted in a significant reduction in skeletal muscle fibrosis (213).

**Figure 5:**
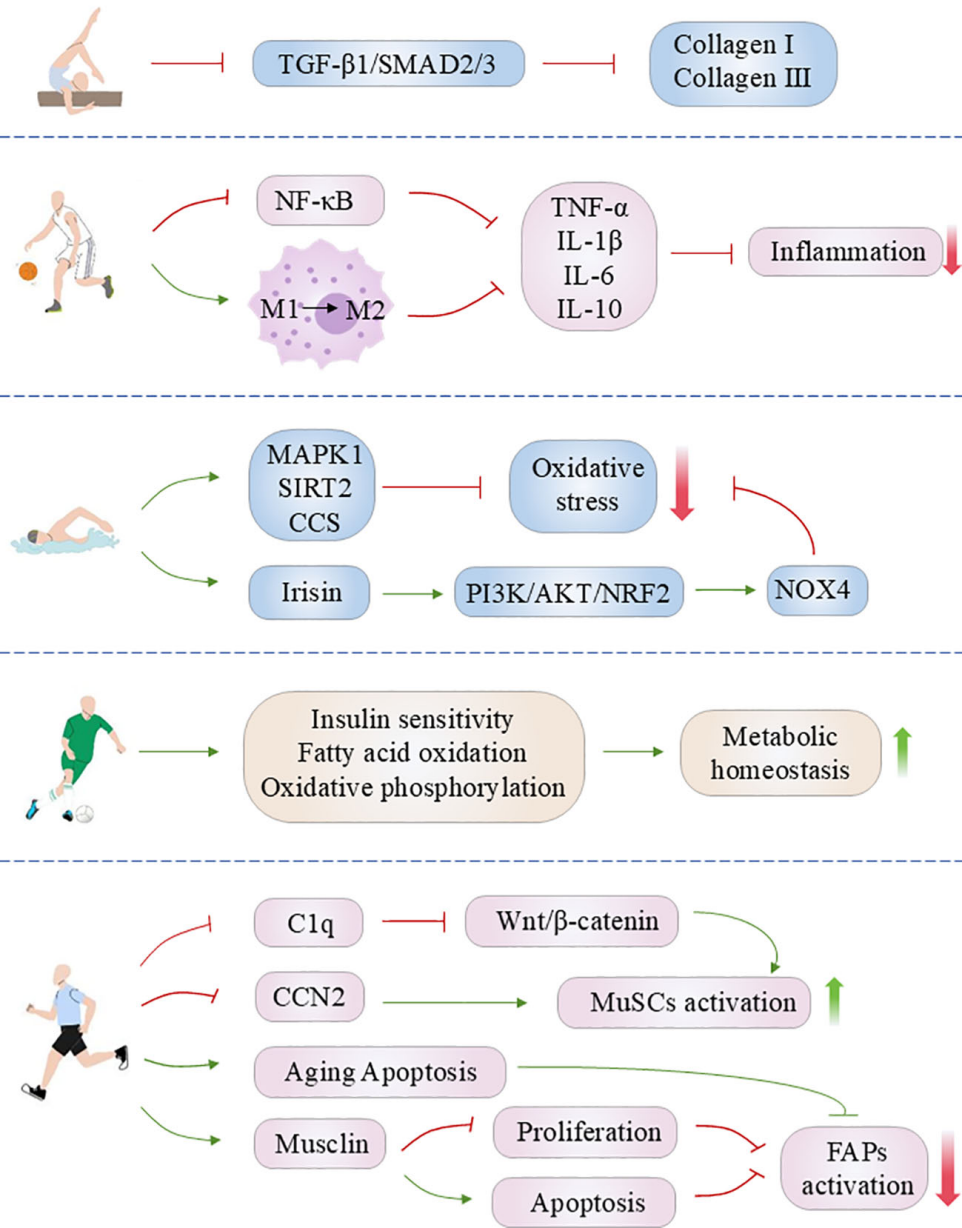
The mechanism by which exercise protects against skeletal muscle fibrosis.

In conclusion, physical activity has been demonstrated to exert a significant anti-skeletal muscle fibrosis effect in a range of pathological contexts. Appropriate exercise effectively slows the development of skeletal muscle fibrosis by inhibiting the inflammatory response, reducing oxidative stress, modulating the TGF-β/Smad signaling pathway, and regulating the activation and function of myosatellite cells and FAP. However, in certain circumstances, exercise may also exacerbate skeletal muscle damage and fibrosis. Therefore, the functional status of the individual engaging in exercise and the suitability of the exercise program may be crucial factors in determining the efficacy of exercise as a therapeutic intervention (**Figure 5**) (**Table 2**).

## 8 Clinical practice and challenges

Although exercise therapy holds significant potential in alleviating organ fibrosis, its effective translation into clinical practice still faces multiple challenges. Firstly, individual physiological differences and exercise tolerance in different patients may impact the effectiveness of exercise interventions. Therefore, personalized exercise prescriptions need to be explored for fibrosis treatment based on the specific conditions of the patients (214). Secondly, the optimization of intervention parameters, such as exercise type, intensity, frequency, and duration, is still unclear. Specifically, how to precisely control exercise load and avoid organ strain and injury caused by overtraining remains a challenge. Thus, the best exercise intervention plan needs to be determined through the use of imaging techniques, biomarkers, physical fitness tests, and dynamic monitoring of patient feedback (214). For high-risk patients with multiple complications or underlying diseases, specific exercise intervention plans should be developed, along with close medical supervision (215). Additionally, during exercise training, patients may resist the treatment due to physical discomfort, insufficient understanding of exercise, or changes in lifestyle, resulting in poor compliance. Therefore, enhancing patient education, providing psychological support, and motivational interventions are necessary to help patients build confidence and develop the habit of exercising (216, 217). In clinical practice, the synergistic effects and potential interactions between exercise and other therapeutic modalities (such as dietary interventions, pharmacotherapy, and surgical treatments) still require the promotion of multidisciplinary collaboration and clinical research to explore the combined effects of exercise with various treatments and optimize combined therapeutic strategies (218). Furthermore, while short-term studies show that exercise can effectively reduce organ fibrosis, the long-term effects and safety of exercise interventions remain unclear. Long-term follow-up clinical studies are needed to ensure the safety and efficacy of exercise therapy in long-term use (219). It is important to note that although the biological effects of exercise have been widely studied, standardizing exercise treatment in clinical practice remains a challenge. Therefore, evidence-based exercise therapy guidelines need to be developed, covering exercise interventions for different diseases and patient populations, to ensure the standardization of exercise therapy. Moreover, multicenter, large-sample clinical trials are required to further validate the effectiveness of these standards (220). Finally, policy support and resource allocation from government bodies, including medical insurance policies, the availability of exercise facilities, and the training of physical therapists, are essential for the widespread application of exercise therapy (221).

## 9 Conclusion and prospect

Tissue fibrosis is a pathological change characterized by the proliferation of large amounts of scar tissue and excessive deposition of ECM within the organ stroma. It typically occurs in the middle and late stages of various diseases. The continued progression of fibrosis leads to the destruction of the organ’s normal tissue structure, ultimately causing a decline in organ function (1, 2). Severe tissue fibrosis can result in organ failure, which is a significant contributor to mortality and represents a substantial challenge to human health. Currently, there is no effective treatment for tissue fibrosis. For patients with end-stage fibrosis, organ transplantation is the only available option. Exercise has been demonstrated to be an economical and relatively safe intervention modality with broad-spectrum targeting and long-lasting effects and has been applied in the comprehensive treatment and management of fibrotic diseases.

This review presents a summary of the most recent research and applied advances in the field of exercise modulation of tissue fibrosis. A growing body of evidence from both clinical and preclinical studies indicates that appropriate exercise has a beneficial effect on the regulation of fibrosis in several organs, including the heart, lungs, kidneys, liver, and skeletal muscle. Furthermore, it has been demonstrated that exercise can slow down or even reverse the progression of tissue fibrosis in organs damaged by a variety of factors, thereby significantly improving their functional capacity. Specifically, Exercise can alleviate fibrosis in various organs by directly inhibiting the activation of fibroblasts, reducing the expression of pro-fibrotic factors such as TGF-β, and slowing collagen deposition. Additionally, exercise can regulate inflammatory pathways such as NF-κB and NLRP3, decreasing inflammatory cytokines and immune cell infiltration to modulate inflammation (62, 101, 117, 184–186). Furthermore, exercise enhances the activity of endogenous antioxidants like SOD, CAT, and GPx, and reduces ROS production, which is particularly important in inhibiting oxidative damage involved in organ fibrosis (69, 101, 123). Exercise also lowers the risk of fibrosis by improving mitochondrial function, enhancing ATP synthesis, and regulating metabolic pathways such as AMPK/Sirt1/PGC-1 (69, 132). Moreover, exercise modulates autophagy through pathways like AMPK and mTOR, promoting the clearance of intracellular waste and cellular repair (15, 134). Beyond metabolic factors, exercise reduces the accumulation of senescent cells and alleviates ECM deposition, thereby improving fibrosis (135). Apoptosis plays a dual role in fibrosis. Exercise can suppress excessive cell apoptosis by regulating caspase and Bcl-2/Bax pathways, while promoting cell repair and regeneration through p53 signaling, thereby inhibiting further fibrosis progression (107, 137, 167). Although these mechanisms are common, there are indeed differences between organs. In myocardial fibrosis, exercise reduces fibroblast proliferation and ECM remodeling by promoting the release of exosomes containing miR-455, miR-29b, miR-323-5p, and miR-466 (88). In pulmonary fibrosis, the impact of airway mechanical deformation is also present. Exercise modulates macrophage and fibroblast function, alleviates pulmonary inflammation, improves lung function, and reduces fibrosis triggered by mechanical stress (18). In renal fibrosis, exercise regulates key molecules in lipid metabolism (such as PPARα) and the renin-angiotensin system, reducing lipid accumulation and slowing the progression of fibrosis (127, 139, 140). Liver fibrosis often accompanies the activation and excessive proliferation of HSCs. Exercise inhibits HSC activation and promotes liver regeneration and repair (14, 172). Additionally, as the central organ for lipid metabolism, the liver is most affected by lipid metabolic disorders. Exercise improves liver lipid metabolism, thus reducing liver fat accumulation and fibrosis (160–162). In skeletal muscle fibrosis, exercise activates MuSCs via the Wnt/β-catenin pathway, alleviating fibrosis caused by injury, and enhances FAP apoptosis, promoting muscle regeneration while reducing collagen deposition and fat infiltration (23, 208, 209). Furthermore, skeletal muscle is the only organ that can be regulated by passive exercise. During passive exercise, muscle tissue is subjected to mechanical stretching, which enhances muscle adaptability and reduces the occurrence of fibrosis (210, 211). However, inappropriate exercise can trigger or accelerate the development of tissue fibrosis, causing damage to the corresponding organs and having detrimental effects on both patients and the general population (142, 200).

To date, numerous studies have revealed the regulatory effects of exercise on tissue fibrosis and its underlying mechanisms, leading to growing belief that exercise has become a promising therapeutic strategy for the prevention and management of fibrosis-related organ damage. However, there are still several challenges and limitations that need to be addressed. Firstly, existing studies have primarily focused on animal models, with a paucity of large-scale clinical trials and long-term follow-up data to verify the actual effects of exercise interventions and their safety. Furthermore, the precise mechanism of action of exercise on fibrosis remains unclear, particularly about fibrosis in different types of tissues. Additionally, there is a need to investigate how to accurately modulate different types and modalities of exercise to respond to the specific needs of each type of fibrosis. In fact, in addition to the aforementioned organs and tissues, fibrosis also occurs in adipose tissue and the intestine, although research on these areas is relatively limited. Adipose tissue fibrosis is one of the key pathological features of metabolic diseases such as obesity (222). Intestinal fibrosis is commonly seen as a long-term complication of inflammatory bowel disease (IBD) (223). Recent studies have shown that exercise can slow down or even reverse the inflammation and fibrosis of adipose tissue induced by HFD in mice (224, 225). A recent clinical study also reported that long-term exercise can affect the ECM composition of subcutaneous abdominal adipose tissue, reducing the abundance of collagen type Col6a, which is associated with metabolic abnormalities, thereby improving the health of adipose tissue (226). These findings provide evidence and mechanistic insights into the role of exercise in regulating adipose tissue fibrosis. Regarding intestinal fibrosis, the beneficial effects of exercise on gut health have been well established (227, 228). There is still a lack of direct and sufficient evidence supporting whether exercise also plays a regulatory role on intestinal fibrosis. Therefore, further investigation into the potential and effects of exercise in regulating fibrosis in specific tissues and organs, such as adipose tissue and the intestine, requires more attention from researchers.

In conclusion, given the numerous advantages that sport offers over traditional drugs and other means, its use as an intervention for fibrotic diseases presents a promising avenue for further research and application. As research progresses, the specific application of exercise intervention in fibrosis treatment is likely to yield enhanced benefits for patients in the future.
